# Generative mathematical modelling to demonstrate virtual simulations of neovascular age related macular degeneration

**DOI:** 10.1371/journal.pone.0189053

**Published:** 2017-12-06

**Authors:** David Hoyle, Tariq Mehmood Aslam

**Affiliations:** 1 Independent Researcher, Manchester, United Kingdom; 2 School of Pharmacy and Optometry, Faculty of Biology, Medicine and Health, University of Manchester, Manchester, United Kingdom; 3 Royal Eye Hospital, NHS Central Manchester University Hospitals, Manchester, United Kingdom; 4 Heriot-Watt University, Edinburgh, United Kingdom; University of Birmingham, UNITED KINGDOM

## Abstract

**Purpose:**

To develop a generative mathematical model of wet age-related macular degeneration (AMD) and model the impact of injections of anti-vascular endothelial growth factor to virtual patients with the condition.

**Methods:**

We isolated key pathophysiological components of macular degeneration in terms of macular edema development and response to anti-vascular endothelial growth factor (VEGF) agents. We developed mathematical models for each of these components using constants determined from published biological experimentation. Consequently, we combined the mathematical models of the separate components to arrive at an end-to-end model of the evolution of macular edema size and its response to treatment.

**Results:**

We present a series of simulations based upon our idealised model. Initially, we demonstrate the theoretical change in macular edema height in wet macular degeneration over time without and with anti-VEGF interventions. In our final simulation, we demonstrate the powerful possibilities of virtual clinical trials by simulating a virtual model of a landmark study using our existing mathematical AMD model.

**Conclusions:**

Using our mathematical modelling based upon known pathological and pharmacological processes we have been able to model the effect of intravitreal injection of an anti-VEGF agent on macular edema from age related macular degeneration. We were subsequently able to mathematically simulate a major clinical trial with results that mirror many key features of the clinical established study. We anticipate that the generative model presented here can evolve to be a useful supportive tool in the challenge to deliver optimal therapy for patients with wet macular degeneration.

## Introduction

Age-related macular degeneration (AMD) is the most common cause of visual impairment and blindness in industrialized nations[1] and the development of choroidal neovascular changes (wet AMD) accounts for more than 80% of associated severe visual disability.[2] Landmark studies have revolutionised care of wet AMD by demonstrating that regular intravitreal anti-vascular endothelial growth factor (anti-VEGF) can deliver far better visual outcomes than have previously been possible.[3, 4] In practice physicians do not treat as rigidly and regularly as in these studies,[5] aiming to reduce potential complications, discomfort to patient and cost. However, a single optimal treatment paradigm remains elusive, with unanswered questions such as optimal dose, frequency and pattern of treatment. Each of these questions would be most accurately determined by clinical trials and many are underway, but they are costly and time consuming and it would not be feasible to assess every possible combination. Physicians are often left to attempt to individualise care by translating limited trial information and incorporating their own understanding of pathology and clinical acumen. Unfortunately, the associated real-world outcomes are currently far inferior to those of the landmark studies[6] and ensuring that treatment decisions lead to optimal outcomes remains a challenge.

Development of a mathematical model for wet AMD could provide a framework to support and augment clinical opinion and trial data. Firstly, it could allow us to construct patient-specific models from which we can determine optimal and individualised treatment regimens tailored to each patient. Secondly, we would gain the ability to run theoretical treatment scenarios, quickly obtaining results for computer-generated virtual clinical trials. This information could not replace clinical trials but might augment clinical opinion where no trial data exists or be used to steer the setup of full clinical trials towards those most likely to provide patient benefit.

In order to construct patient-specific models or run virtual trial scenarios it could be possible to take a machine-learning approach to the problem using a generic model or algorithm to ‘learn’ from patient data. This has some advantages in that it would be unconstrained by existing dogma and is not as dependent on domain expertise, allowing it to yield novel insights. However, the generic and highly flexible mathematical forms used in machine-learning models, such as deep-learning neural-networks, typically require a large number of training data points, possibly far in excess of the number of observations clinically available. Similarly, with regard to virtual clinical trials a machine learning approach could be limited in determining the effects of any intervention outside of the range of training sample data.

Robust, interpretable, and credible extrapolations may come from a generative model; one that has been constructed on the basis of existing biomedical knowledge of the condition and the relevant physiological and biochemical processes. In this paper, we develop a generative mathematical model that is appropriate to age related macular degeneration with the modest initial aim of modelling the impact of injections of anti-VEGF to a newly diagnosed virtual patient with wet age related macular degeneration. We then demonstrate the capacity to run realistic virtual trial scenarios by incorporating relevant aspects of patient-to-patient variation.

## Methods

### Principles of model

**Fig 1:**
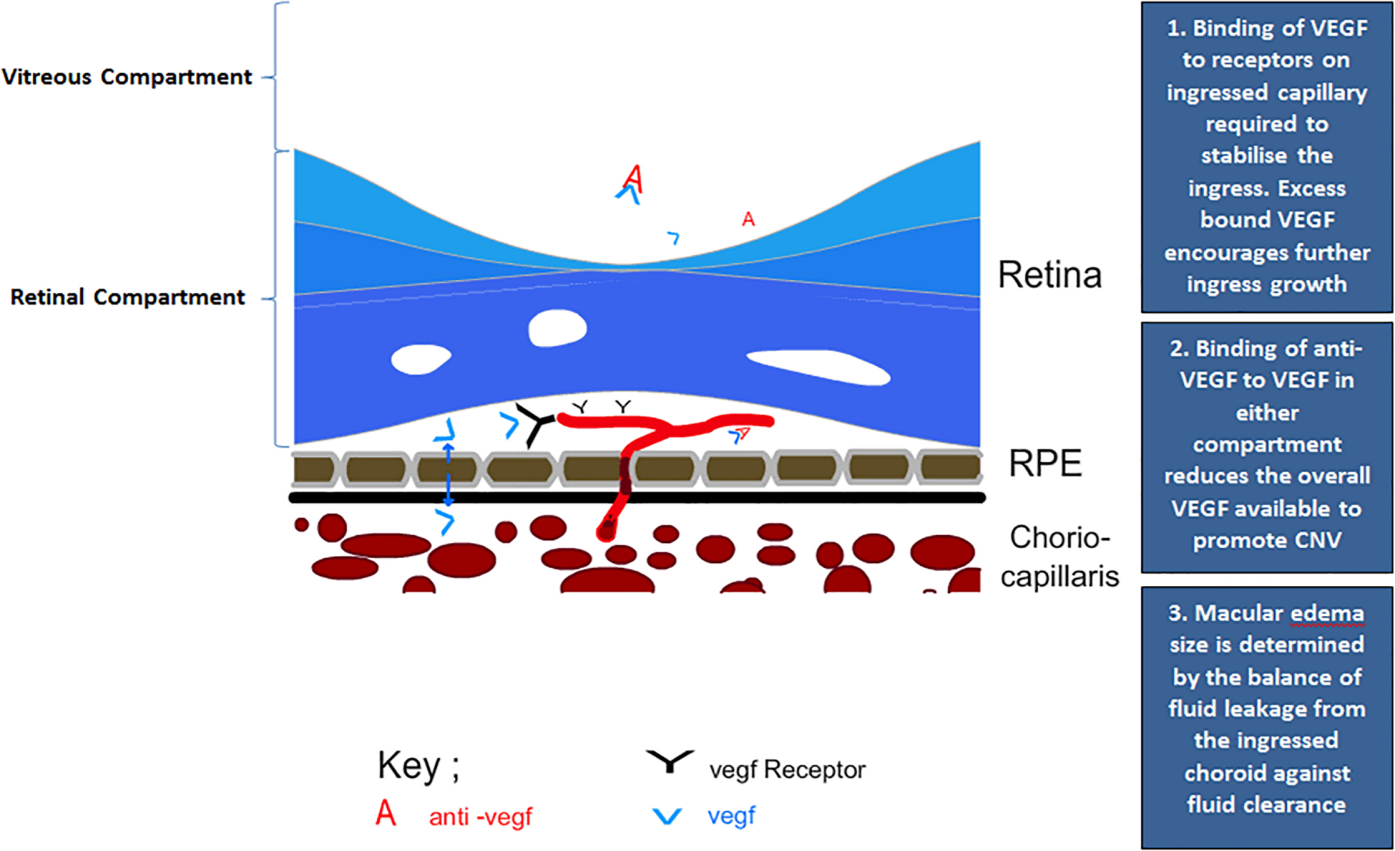
Schematic showing the major clinical aspects determining macular edema size in sub-retinal wet AMD. In this diagram we have stylised the fundamental processes underlying neovascular membrane development with regard to the key components of VEGF, VEGF receptors and anti-VEGF. The description to the right explains the sequence of processes involved.

For our modelling we have attempted to capture as much known pathology as possible, with variables and constants determined from separate biological experimentation wherever possible. However, modelling of biological systems invariably requires some pragmatic simplification of known processes and some level of idealisation. We model the macular edema associated with a classic age-related sub-retinal choroidal neovascular membrane. Fig 1 demonstrates how the region around the macular edema that we are interested in modelling naturally splits into two distinct compartments, and so we develop a two-compartment model. We refer to the first, vitreous compartment as the body of vitreous where anti-VEGF agents are introduced into the system. The second, retinal compartment refers to the deeper portion from intra-retinal and sub-retinal macular edema to the part of the choriocapillaris where the neovascular ingress has originated from, or is at risk of originating from. As with any compartment model, we need only capture the major characteristics of each compartment, and so we model them with idealized geometries. For the vitreous compartment we use a spherical geometry, of radius *R*. For the retinal compartment we are considering only a small part of tissue near to the macular edema, and so to first approximation we can ignore any curvature and use a rectangular geometry.

The scenario in Fig 1 also illustrates that VEGF levels are determined by a number of factors. From this we have determined that our mathematical model will need to consist of five main components,

1. A model that determines the rates at which new VEGF and anti-VEGF are introduced into the system.
2. A model that determines and links the level of VEGF and the level of anti-VEGF.
3. A model that relates the levels of VEGF and anti-VEGF to the level of bound VEGF receptors.
4. A model that relates the level of bound VEGF receptors to the rate of growth of ingressed neovascular choroid.
5. A model that relates the amount of ingressed choroid to the rate of growth of the macular edema.

**Fig 2:**
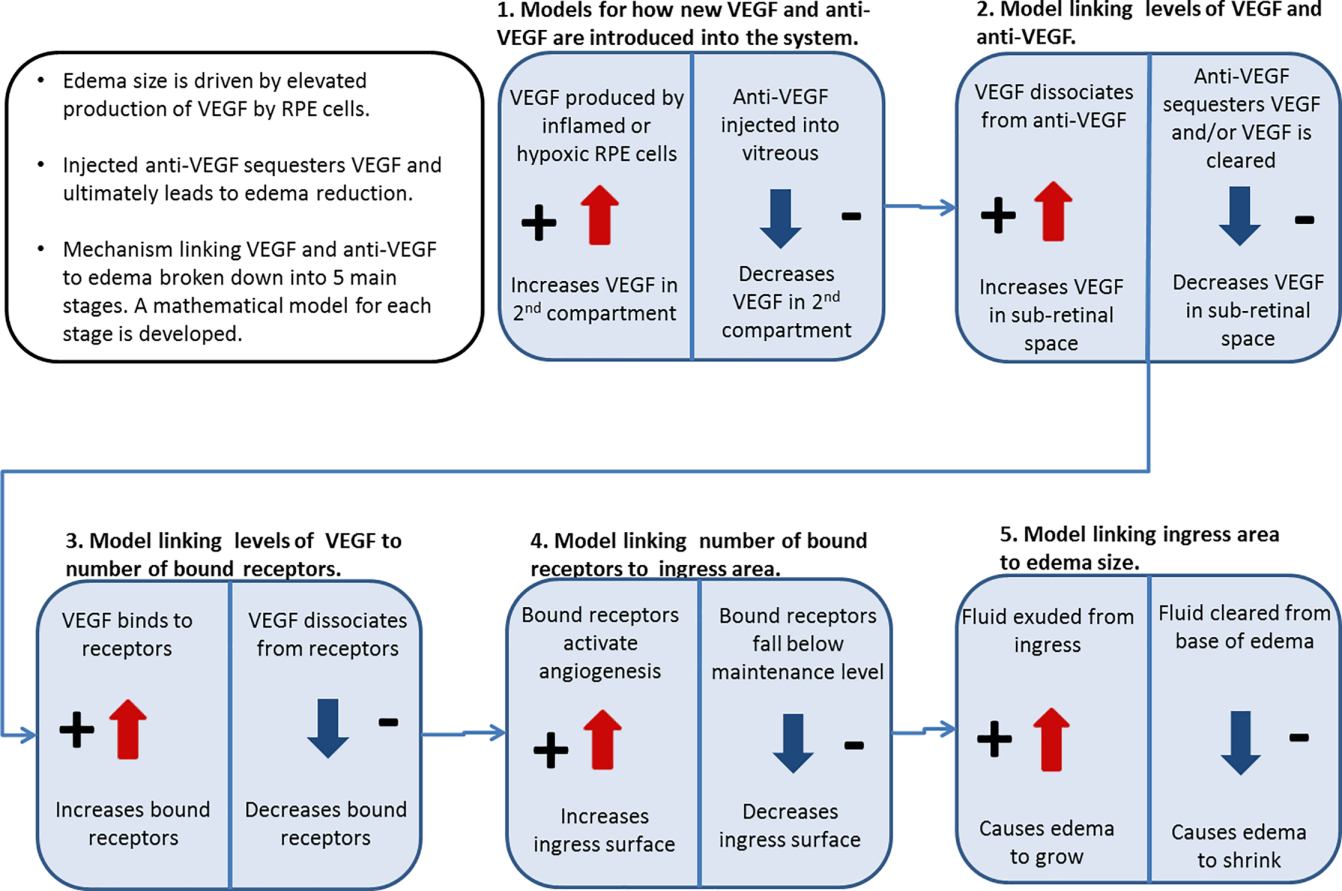
Schematic showing the main mathematical model components linking VEGF and anti-VEGF levels to macular edema size. Separating the complex process of response of neovascular tissue to anti-VEGF agent into these components allows us to focus on algorithms to describe each aspect, before combining to produce an overall model.

We will develop simple mathematical models for each of these five components in relation to the two principal compartments. Each model component serves as the input into the next model component. Consequently, we combine the models of the separate components to arrive at an end-to-end model of the evolution of macular edema size and its response to treatment. This is summarized schematically in Fig 2.

The five component mathematical models are summarized below, where we give the main relevant mathematical facets of each model. The full mathematical details of each component model and the choices of parameter values are given in the supporting information. The five component models lead to a set of coupled differential equations which we solve numerically using the deSolve package[7] for the R statistical computing language.[8]

### Component model descriptions

1. A model that determines the rates at which new VEGF and anti-VEGF are introduced into the system

Anti-VEGF agent is introduced into the vitreous compartment via a series of injections of specified volume, causing a stepped increase in the anti-VEGF concentration.

In the retinal compartment we have direct production of VEGF but no direct introduction of anti-VEGF. Our model of VEGF production in the retinal compartment has two main aspects–up-regulated and basal RPE cells. Healthy basal RPE cells within the macular area produce VEGF at a background rate of ν_0_. In AMD the basal RPE cells become up-regulated, for example due to hypoxia within the RPE induced by thickening with age of the Bruch’s membrane[9] or due to oxidation induced inflammation.[10] Up-regulated RPE cells produce VEGF at an elevated rate of ν_1_ > ν_0_. Suitable values of ν_0_ and ν_1_ are determined by matching model predictions for the typical vitreal concentrations of VEGF in unaffected and affected patients to clinically observed concentrations (see the supporting information for details).

Our model needs to acknowledge established clinical observations that over time individuals with wet macular degeneration often require less frequent treatment to control exudation. The exact mechanism for this is not known, but the principle is incorporated by assuming that once an up-regulated RPE cell has exited its up-regulated state it expresses VEGF at a lower rate. After specifying the rates at which RPE cells enter and leave an ischemic state, the simplifications outlined above lead to a set of differential equations (see supporting information) which are easily solved to obtain the rate of production of VEGF within the retinal compartment.

2. A model that determines and links the level of VEGF and the level of anti-VEGF.

In addition to production, in order to derive the levels of VEGF in the retinal compartment, we must consider other processes;

- Binding of VEGF and anti-VEGF, and corresponding dissociation of existing VEGF + anti-VEGF complexes.
- Clearance of VEGF, anti-VEGF, and VEGF + anti-VEGF complexes

We model the binding and dissociation processes using standard second order reaction kinetics (see supporting information for details). This requires appropriate values for the various binding and dissociation constants, which we take from a number of literature sources. [11–13]

To set clearance rates of VEGF and VEGF+anti-VEGF complexes from the first compartment we assume, following Stefanini et al.,[11] that clearance rates are inversely proportional to molecular size, which in turn is proportional to molecular weight. Knowledge of the molecular weights thus allows us to express the clearance rate of VEGF + anti-VEGF complexes in terms of the clearance rates of VEGF and anti-VEGF from the vitreous compartment (see supporting information for details). We also consider the rates at which the various molecular species enter or are cleared from a compartment to be proportional to the surface area across which the transport is occurring.

Combining the contributions from production, binding, dissociation, and clearance, we obtain a set of differential equations controlling the evolution of the compartmental concentrations of the various molecular species, pertinently of VEGF in the retinal compartment. The differential equations are solved numerically.

3. A model that relates the levels of VEGF and anti-VEGF to level of bound VEGF receptors

Having derived equations that allow us to determine the levels of VEGF in the retinal compartment, we now determine its impact on neovascularisation by deriving formulae for binding (and corresponding dissociation) of VEGF to VEGF receptors on the surface of the ingressed neovascular choroid and existing choriocapillaris.

In order to derive number of bound VEGF receptors, we first require an expression of the total number of receptors. This is given by,

$$\mathrm{Total}\;\mathrm{number}\;\mathrm{of}\;\mathrm{VEGF}\;\mathrm{receptors}=\sigma_{R}\times \left(C_{0}+C_{CNV}\right)$$

where σ_*R*_ is the receptor density (per unit area) which we derive **f**rom Imoukhuede and Popel.[14] Here *C*_0_ is the capillary surface area of the existing choriocapillaris within the macular retinal compartment, and *C*_*CNV*_ denotes the surface area of the ingressed neovasculature. We set a value for *C*_0_ by considering a typical cross-section of the retinal compartment (see supporting information for details) and relating *C*_0_ to the average capillary radius, *R*_*C*_, within the choriocapillaris, and the typical cross-sectional packing fraction, *PF* (how much of the cross-section image the choriocapillaris appears to take up). We take *R*_*C*_ = 40μm, and set *PF* = 0.4, based upon our own assessment of Fourier-domain optical coherence tomography imaging studies.

With knowledge of VEGF concentrations in the retinal compartment and of total number of receptors, we can use standard second order reaction kinetics to construct a differential equation determining the level of bound VEGF receptors. As before, we solve this differential equation numerically in conjunction with the other differential equations.

4. A model that relates the level of bound VEGF receptors to the rate of growth of ingressed choroid.

We have modelled above the interactions of VEGF, anti-VEGF and receptors including development of functions that describe levels of bound VEGF. This is the direct stimulus for causing growth of choroidal tissue. We examine now exactly how this bound VEGF leads to abnormal choroidal growth.

As the mechanism linking VEGF receptor signalling to the neovascularization process has not been fully elucidated, we propose a heuristic model for the choroid growth, whereby the rate of growth of *C*_*CNV*_ is simply proportional to the total signal transduced by bound VEGF receptors, and therefore proportional to the number of bound VEGF receptors in the retinal compartment. As a background level of VEGF is required even to maintain the choriocapillaris under normal conditions,[15, 16] we make the rate of growth of *C*_*CNV*_ proportional to the level of bound receptors above a specified threshold. So the simplest model we can propose takes the form,

$$\mathrm{Rate}\;\mathrm{of}\;\mathrm{growth}\;\mathrm{of}\;C_{CNV}=a_{1}\times \mathrm{Number}\;\mathrm{of}\;\mathrm{growth}\;\mathrm{stimulating}\;\mathrm{receptors}\times \left[\frac{\mathrm{Bound}\;\mathrm{fraction}\;\mathrm{of}\;\mathrm{receptors}}{a_{2}}-1\right]$$

The parameter *a*_1_ determines the rate of choroid growth for a unit level of stimulus (determined by matching with observed clinical timescales for macular edema growth). The parameter *a*_2_ is the threshold level of bound VEGF receptors that is required before new choroid growth occurs.

We know that in certain conditions neovascularization is inhibited within the region above the choriocapillaris by factors such as TIMP-3 and PEDF.[17, 18] As VEGF and CNV levels increase, such inhibitory factors are upregulated.[19, 20] We incorporate these experimental findings by making *a*_2_ dynamic, increasing with increasing macular edema size.

The number of growth stimulating receptors in the retinal compartment we take to be less than the total number of VEGF receptors, and we set this equal to σ_*R*_ *×* (*b*_0_*C*_0_ + *C*_*CNV*_). The parameter *b*_0_ is between 0 and 1, and represents the fact that although VEGF receptors will be bound throughout the entire macular choroid area within the retinal compartment, only a small fraction of *C*_0_ will be capable of contributing to the new growth–namely that portion of the existing choroid that is capable of breaking through the Bruch’s membrane and RPE into the sub-retinal space. Clearly *b*_0_ cannot be so insignificantly small so as to preclude growth. Equally, observations from clinical images indicate *b*_0_ should not be a large proportion of available macular choriocapillaris. For this study we set *b*_0_ = 0.1.

5. A model that relates the amount of ingressed choroid to the rate of growth of the macular edema.

We now turn our attention to describing a model that relates the amount of ingressed choroid to the consequent rate of growth of the macular edema volume, *V*_*edema*_. As macular edema base area *S*_*edema*_ and macular edema height are more easily clinically measurable we focus upon these characteristics. Growth of the macular edema is due to increased amounts of fluid leaking from the newly stimulated ingressed choroid. We assume the rate of fluid leakage from the ingressed choroid is proportional to the surface area of the ingress. This gives us a term for the rate at which fluid enters the macular edema. Similarly, we assume that fluid from the ingressed choroid is ultimately cleared through channels within the RPE layer. We assume the density of channels in the RPE through which fluid clearance can occur is approximately constant and thus the rate of clearance is proportional to the base area of the macular edema. The rate of growth of the macular edema volume, *V*_*edema*_, is then simply the difference between the rate at which fluid enters the macular edema and the rate at which fluid is cleared from the macular edema (we consider the leaked fluid to be incompressible). It then only requires us to mathematically relate the macular edema volume *V*_*edema*_ to the macular edema base area *S*_*edema*_ to obtain a mathematical model for the rate of growth of macular edema height. This is calculated using simple trigonometry assuming radial symmetry of the macular edema and an approximation of constant contact angle–the angle within the edema formed between the sub-retinal surface and the RPE layer. An appropriate value for the contact angle is derived from clinical OCT data.

## Results

**Fig 3:**
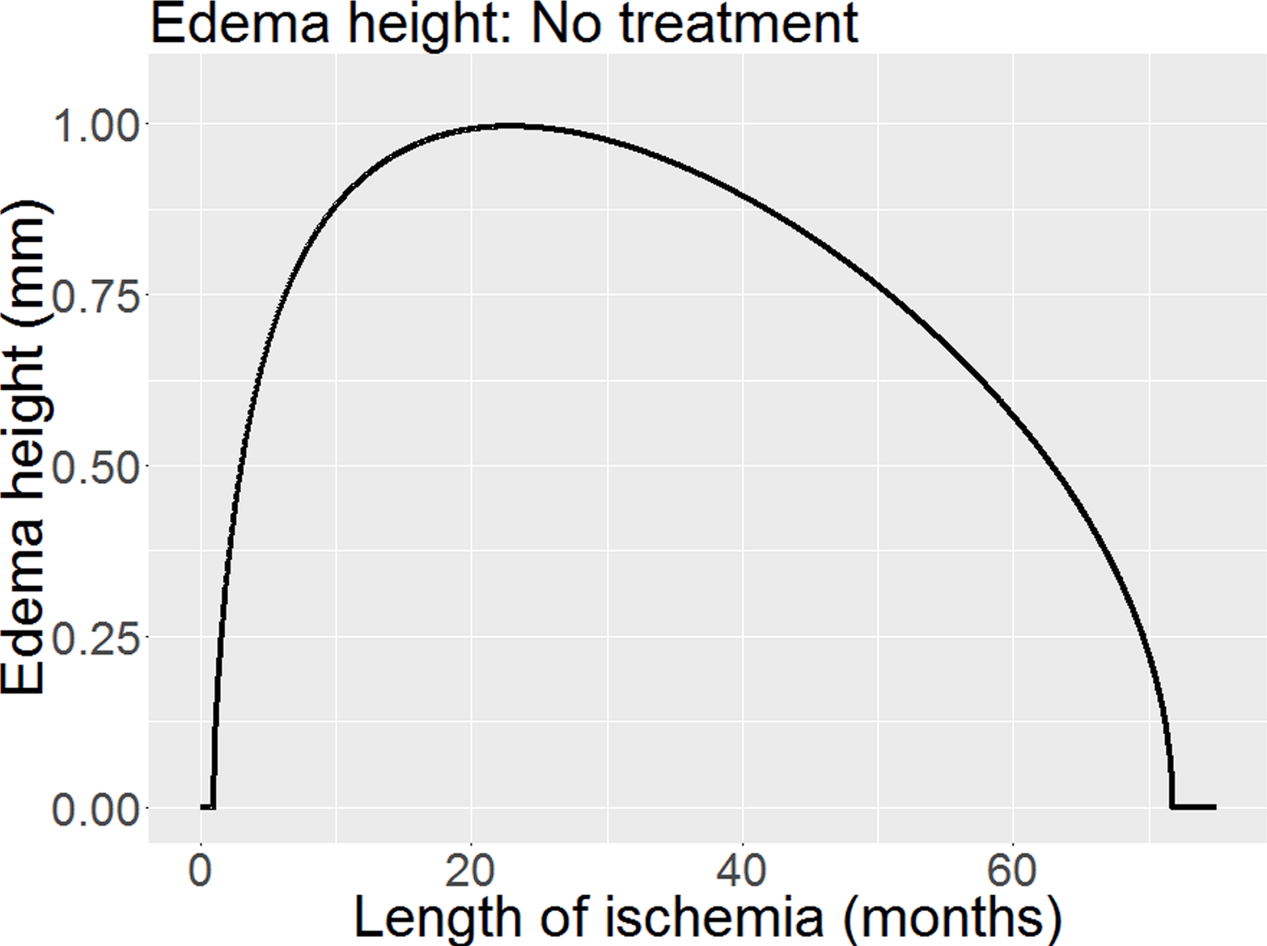
Macular edema height changes in stylised Wet AMD model with no intervention. This is a theoretical model with outcomes that are not apparent in clinical practice due to the usual need for prompt treatment of wet AMD.

In our first simulation, shown in Fig 3, we demonstrate the theoretical change in macular edema height in our idealised model of wet macular degeneration over time without any intervention. We note key features of an early rapid rise in macular edema height followed by a reducing rate of growth and ultimate regression over some years.

**Fig 4:**
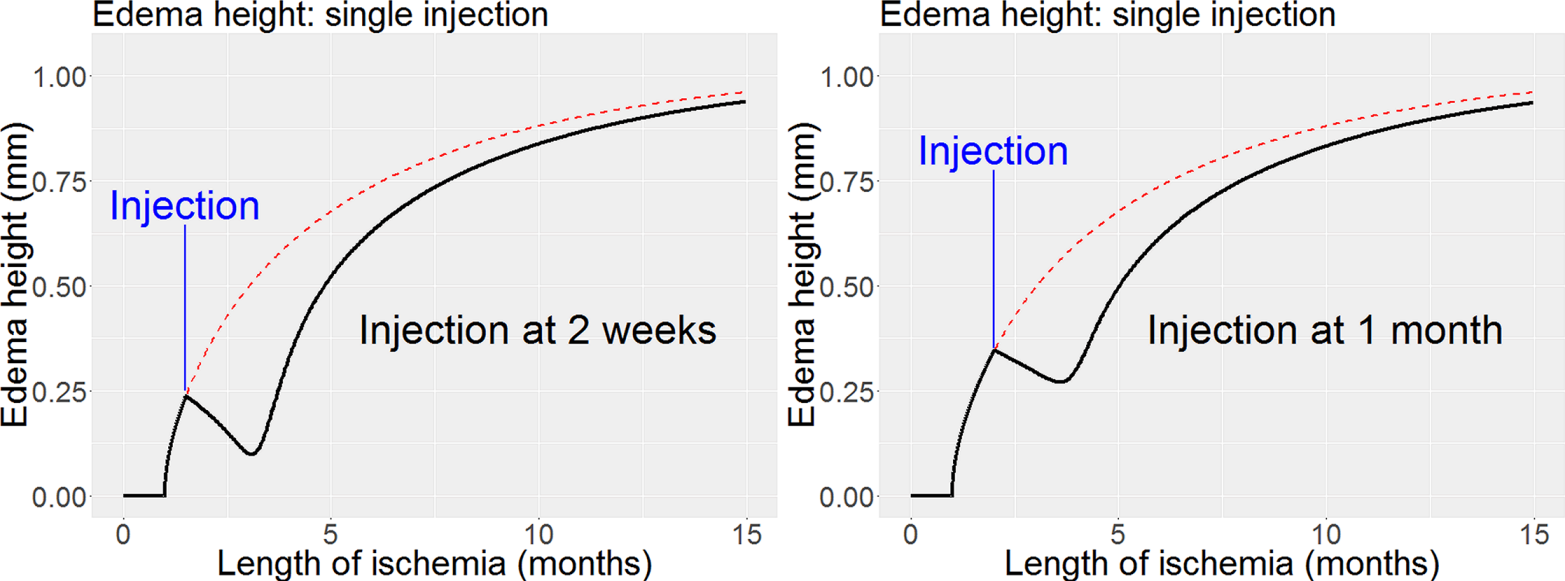
Impact of anti-VEGF injection (aflibercept) at two different time periods following onset of exudation from Wet AMD. In the left graph injection is given at 2 weeks after onset of the macular edema and in the right graph at one month post onset. In both graphs the dashed red line indicates the trajectory of the edema height in the absence of any injections. This schematic represents the artificial situation of treatment with a single injection only, but demonstrates clearly the greater impact when this first injection is delivered earlier, and also the need for continued treatment for long term gain.

In our second simulations, shown in Fig 4, we model the impact of a single 2mg injection of Eylea (aflibercept) into a virtual patient with newly developing Wet AMD. In the first of these injection simulations the injection is administered early at 2 weeks after onset of exudation. The impact of the injection is to cause a steady decrease in macular edema height which then begins to increase again in height. If the same injection is given after a slightly greater initial delay, at 4 weeks after onset of exudation, the impact is different. Firstly the rate of decrease in height of macular edema is reduced. Secondly the absolute and percentage reduction in macular edema height caused by giving the injection is reduced.

**Fig 5:**
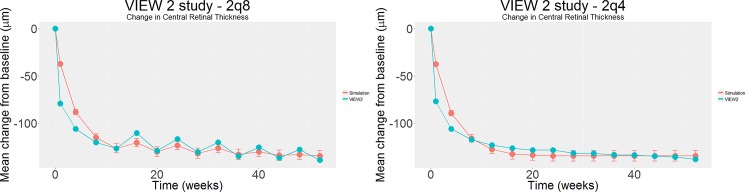
Computer generated plot of the predicted mean change in macular edema height over time, in samples with the same baseline characteristics as the corresponding arm of the VIEW 2 study. The plots generated by the nascent mathematical model are superimposed, along with error bars (+-2S.E), on the actual results of the human VIEW 2 clinical trial. Left-hand plot shows the ‘2q8’ arms, whilst the right-hand plot shows the ‘2q4’ arm.

In our final simulation, shown in Fig 5, we address and demonstrate the possibilities of virtual clinical trials. We simulate a virtual model of the ‘2q8’ and ‘2q4’ arms of the landmark VIEW 2 study [21] using our existing mathematical AMD model to generate results for a large group (n = 1000) of patients whose mean central retinal thickness at presentation is matched to the value of the corresponding arm of the actual VIEW 2 study. Thus, the baseline information on participants in the real trial was included in setting up the analysis, but subsequently no information from the trial was added. We note that there is a rapid reduction in mean change in macular edema height and this initial rapid reduction slows down with time. Macular edema height still shows gradual reduction overall, but in the ‘2q8’ arm oscillates somewhat such that a visible waveform is apparent. This waveform of oscillation decreases with amplitude over time.

## Discussion

We are acutely aware at the outset of this study that deficiencies exist in all computer models, including our own, when predicting complex systems be they natural events such as weather or biological systems. These models would never replace real world experimentation or clinical trials. The model we present here only incorporates a very simple model of generalised AMD pathogenesis as far as we are able to understand it with current literature. It contains estimations based upon experimental and clinical data and we make many idealised assumptions.

We present only a very first prototype with results of some basic tests. We challenged it by modelling a recreation of a well-known clinical trial which had many specific and defining features. Our recreation was entirely generated through use of the equations listed in the methods section of this paper. We did not attempt to modulate the equation parameters to obtain a good fit with the VIEW 2 study but based them on general biological principles explained in this paper. Given this and the levels of variability in any given AMD patient population, we would not expect complete agreement of outcomes with VIEW 2. However, the resultant output does indeed mirror many of the key features found in the VIEW 2 study.

Our current mathematical model has 20 parameters that have been derived directly from scientific texts such as size of various molecules and dissociation constants. It also involves around 10 parameters that would potentially be amenable to allowing simulations to be run with different molecules and allowing for inter-individual differences that are so apparent in clinical practice, with the potential ultimately for individualised treatment simulations. Treatments could be simulated to be given as single injections, regular two-monthly injections, injections only given with certain macular edema size at monthly visits (PRN), or treat and extend injections.

We have presented here the first results of our prototype model and described the basic features of it that allow simulation of clinical trials.

Further clinical validation of this model is crucial. It will be necessary to compare and analyse the results of the simulations quantitatively against experimental evidence from real patient responses and this is planned for future studies. This step will be essential to provide further validation but also to set a benchmark in view of comparing future versions of the model and identifying improvements.

Indeed, we anticipate our model will become more sophisticated and realistic over time, incorporating further scientific, clinical and biological data. Its accuracy will improve with each update. After further clinical validation we expect to be able to run simulations to compare different treatment regimens with different anti-VEGF agents. We also hope to be able to derive mathematical values for a wide range of user specific variables from real patients and to use these variables to predict their own progression of wet macular degeneration and the best combination of drugs and treatment regimens to control it. Whilst never replacing information from clinical trials or clinical acumen, we hope that the generative model presented here can evolve to be a useful supportive tool in the challenge to ensure optimal therapy for patients with wet macular degeneration.

## Supporting information

S1 File Detail of mathematics and model construction. (DOCX)
