# Supplementary material for: Generative mathematical modelling to demonstrate virtual simulations of neovascular age related macular degeneration
Source: PLoS One. 2017 Dec 6;12(12):e0189053. doi: 10.1371/journal.pone.0189053 (PMC5718607; doi:10.1371/journal.pone.0189053)
Supplement: S1 File — (DOCX) [file pone.0189053.s001.docx]

Supporting Information

Within this supplementary information we give full details of each of the component mathematical models that have been introduced in the main text. We denote the concentrations of VEGF, anti-VEGF, and VEGF+anti-VEGF complexes by $y_{1}^{(c)}$, $y_{2}^{(c)}$*,* and $y_{3}^{(c)}$respectively. The superscript, $c=1,2$, denotes the compartment.

# A model that determines the rates at which new VEGF and anti-VEGF are introduced into the system

## Compartment 1 (Vitreous):

**Production of anti-VEGF**

Within the first compartment production is only relevant for anti-VEGF and occurs via injection. We consider the injected anti-VEGF to diffuse within the compartment relatively instantaneously and the rate of change in the concentration of anti-VEGF due to a series of individual injections is modelled as a series of Dirac delta-functions. That is, the contribution to the rate of change of anti-VEGF in the first compartment is $f_{2}(t)$, and is given by,

$$f_{2}\left( t \right) =\sum_{i=1}^{N_{Injection}} v_{I}^{(i)}\delta\left( t- t_{I}^{(i)} \right) \text{Eq.(S.1) }$$

where the $N_{Injection}$ injections are given at times $t_{I}^{(i)}, i=1,\cdots, N_{Injection}$ , with amounts $v_{I}^{(i)}$. For bevacizumab injections we set $v_{I}^{(i)}$ to be 1.25mg, so that immediately after an injection we approximate that the vitreal concentration of anti-VEGF increases by 1.25mg/vitreal volume. For standard aflibercept injections we set $v_{I}^{(i)}$ to be 2mg.[^1^](#_ENREF_1)

## Compartment 2 (Retinal)

In the second compartment we have direct production of VEGF but no direct introduction of anti-VEGF.

**Production of VEGF**

Our model of VEGF production has two main components – up-regulated and basal RPE cells. Healthy basal RPE cells within the macular area produce VEGF at a background rate of $v_{0}$. The basal RPE cells become up-regulated, for example due to hypoxia within the RPE induced by thickening with age of the Bruch’s membrane, [^2^](#_ENREF_2) or due to oxidation induced inflammation. [^3^](#_ENREF_3) Up-regulated RPE cells produce VEGF at an elevated rate of $v_{1}> v_{0}$ . We consider Basal RPE cells to become up-regulated at a constant rate $\tau_{1}^{-1}$ per unit time, whilst up-regulated RPE cells exit their up-regulated state at a constant rate $\tau_{2}^{-1}$ per unit time.

Once an up-regulated RPE cell has exited its up-regulated state we assume that it expresses VEGF at a rate $\phi v_{0}$, with the parameter $\phi$ being between 0 and 1. By allowing for the possibility of setting a value of $\phi$ that is less than 1 we allow for the possibility that the cumulative effects of dis-regulation of the VEGF/PEDF ratio will result in a new equilibrium state for the RPE cells and hence a lower VEGF basal expression rate. In the absence of any current appropriate literature or clinical data we have for the simulations in the main text set $\phi=0$, so that expression of VEGF is completely suppressed once an RPE cell exits an ischemic state.

The starting population of healthy basal RPE cells will be finite and proportional to the macular area. We denote the number of normal, healthy RPE cells at time $t$, by $N_{RPE}(t)$. Similarly, we denote the number of up-regulated RPE cells at time $t$, by $I_{RPE}(t)$. The time-point $t=0$ corresponds to the point at which we first see RPE cells becoming at risk of upregulated VEGF production. However, as neovascularization and ingress will take some time to occur after the start of VEGF upregulation in the RPE, we include a lag, $\tau_{lag}$, between the onset of VEGF upregulation and the onset of edema growth. For the simulations shown in the main text we set $\tau_{lag}$= 1month. Throughout this work we have defined a month to be equivalent to 30 days. Given these considerations, the dynamics of our VEGF production model, as described above, are captured in the following system of differential equations,

$$\frac{dN_{RPE}(t)}{dt}= -\tau_{1}^{-1}N_{RPE}\left( t \right) \text{Eq.(S.2) }$$

$$\frac{dI_{RPE}(t)}{dt}= \tau_{1}^{-1}N_{RPE}\left( t \right)- \tau_{2}^{-1}I_{RPE}\left( t \right) \text{Eq.(S.3) }$$

These two differential equations can easily be solved, with starting values, $N_{RPE}\left( t=0 \right)= N_{RPE}\left( 0 \right)$, and $I_{RPE}\left( t=0 \right)=0$. We obtain,

$$\text{Rate of VEGF production = }v_{0}\left( 1- \phi\right)N_{RPE}\left( 0 \right)e^{\frac{-t}{\tau_{1}}}+ v_{1}\left( 1- \phi\right)N_{RPE}\left( 0 \right)\frac{\tau_{2}}{\tau_{2}- \tau_{1}}\left[ e^{\frac{-t}{\tau_{2}}}-e^{\frac{-t}{\tau_{1}}} \right] + \phi v_{0}N_{RPE}\left( 0 \right) \text{Eq.(S.4) }$$

This rate of production of VEGF within the second compartment is denoted by $f_{1}(t)$, for use in later models.

To comply with credible clinical timescales we have chosen to set $\tau_{1}=6$ months and $\tau_{2}=12$ months. The values of $v_{0}$ and $v_{1}$ are learnt from clinical data, i.e. they are determined by matching the model output to clinical observations. The value of $v_{0}N_{RPE}(0)$ is determined by matching the pre-edema equilibrium concentration of VEGF in the 1^st^ compartment (vitreous) to the typical vitreous concentration of VEGF within unaffected patients. Similarly, $v_{1}N_{RPE}(0)$ is determined by matching the typical clinically observed vitreous concentration of VEGF within AMD affected patients to the typical vitreous VEGF concentration predicted by the model during growth of the edema. We take the typical modelled vitreous VEGF concentration during edema growth to be the average of the vitreous VEGF concentration when the untreated edema reaches its maximum size and the vitreous VEGF concentration when the edema has reached what we consider to be the smallest typical size at which a patient might first present. This smallest clinically presentable edema size we take to have an edema base area of half the optic disc area. We take the typical or reference optic disk size, $S_{Disc}$, to be 2.4mm^2^ , assuming a typical horizontal disc diameter just under 1.77mm [^4^](#_ENREF_4) and planar disc geometry (to first approximation).

Ascertaining typical clinically observed vitreous VEGF concentrations in affected and unaffected patients is difficult, with few studies directly measuring the vitreous concentrations. Instead, we note that Meyer and Holz [^5^](#_ENREF_5) quote baseline (prior to treatment with bevacizumab) aqueous humour VEGF concentration values of 85 pg/ml within vein occlusion patients,[^6^](#_ENREF_6) and 102.6±90.6 pg/ml for patients with CNV secondary to AMD. Noma et al. [^7^](#_ENREF_7) have shown that aqueous humour VEGF concentrations are comparable to, but typically smaller than vitreous humour VEGF concentrations, though the two are strongly correlated. Consequently, we take 100pg/ml as a not unreasonable figure for the typical VEGF concentration within the first compartment during the growth phase (prior to treatment). Similarly, after bevacizumab treatment aqueous humour VEGF concentrations drop to 18.3±22.5 pg/ml in patients with CNV secondary to AMD. Consequently, we take 10pg/ml to be a representative vitreous VEGF concentration in unaffected patients.

# A model that links the level of VEGF and the level of anti-VEGF.

## Compartment 1 (Vitreous):

The levels of VEGF and anti-VEGF are linked by binding, dissociation and clearance from and into the compartment, and by levels of VEGF+anti-VEGF complexes. These processes are modelled below.

**Binding and Dissociation**

Binding and dissociation occurs between VEGF and anti-VEGF in the first (vitreous) compartment. The amount of binding and dissociation alters the levels of VEGF concentration, anti-VEGF concentration and VEGF+anti-VEGF complex concentration.

In order to model this, we require values for the forward binding constant, $d_{1}$, that determines the rate of formation of VEGF+anti-VEGF complexes, and also the dissociation rate, $d_{-1}$, for dissociation of VEGF+anti-VEGF complexes. For bevacizumab we can follow Stefanini et al.[^8^](#_ENREF_8) and use $d_{1}= {9.2\times10}^{4}\text{L}\text{Mol}^{-1}\text{s}^{-1}$ , $d_{-1}= {2\times10}^{-4}\text{s}^{-1}$. For aflibercept, the picture is less clear. Papadopoulos et al. [^9^](#_ENREF_9) have measured $d_{1}= {410\times10}^{5}\text{L}\text{Mol}^{-1}\text{s}^{-1}$, $d_{-1}={2.01\times10}^{-5}\text{s}^{-1}$ for aflibercept binding VEGF_165_ , and $d_{1}= {1.6\times10}^{5}\text{L}\text{Mol}^{-1}\text{s}^{-1}$ , $d_{-1}={0.73\times10}^{-5}\text{s}^{-1}$ for ranibizumab binding VEGF_165_. However, a forward binding constant for aflibercept that is many times that of ranibizumab would not appear to be compatible with trial study findings. In particular, comparison of results from the ‘Rq4’ and ‘0.5q4’ arms of the VIEW 2 study [^1^](#_ENREF_1) suggest that the efficacy of aflibercept cannot be that dissimilar from ranibizumab, possibly even weaker given that ranibizumab is less than half the molecular weight (48 kDa) of aflibercept (115 kDa) and so should be cleared faster. A more detailed comparison of anti-VEGF agent binding constants has been carried out by Yang et al.[^10^](#_ENREF_10) The values for $d_{1}$ and $d_{-1}$ for aflibercept measured by Yang et al. varied enormously depending upon the Biacore measurement assay format, varying from${3.1\times10}^{5}\text{L}\text{Mol}^{-1}\text{s}^{-1}$ to ${1630\times10}^{5}\text{L}\text{Mol}^{-1}\text{s}^{-1}$ for $d_{1}$, and from ${28.3\times10}^{-5}\text{s}^{-1}$ to ${5906\times10}^{-5}\text{s}^{-1}$ for $d_{-1}$. Measurements of $d_{1}$ and $d_{-1}$ for ranibizumab are more consistent across the assay formats. More critically, Yang et al. observe that ranibizumab is able to displace aflibercept in competitive binding assays, whilst aflibercept is not able to displace ranibizumab. These observations, in conjunction with the VIEW 2 study results, suggest that $d_{1}$ and $d_{-1}$ values similar or weaker than those for ranibizumab would be more appropriate for aflibercept. Consequently, we have set $d_{1}= {10\times10}^{5}\text{L}\text{Mol}^{-1}\text{s}^{-1}$ , $d_{-1}= {1\times10}^{-5}\text{s}^{-1}$ for aflibercept binding VEGF.

Given values for $d_{1}$ and $d_{-1}$ the contributions to the rate of change in concentration of VEGF, anti-VEGF and VEGF+anti-VEGF complexes factoring in the binding and dissociation complexes are,

$$- d_{1}y_{1}^{\left( 1 \right)}y_{2}^{\left( 1 \right)}+ d_{-1}y_{3}^{\left( 1 \right)}\text{ contribution to rate of change of VEGF concentration}$$

$$- d_{1}y_{1}^{\left( 1 \right)}y_{2}^{\left( 1 \right)}+ d_{-1}y_{3}^{\left( 1 \right)} \text{ contribution to rate of change of anti-VEGF concentration}$$

$d_{1}y_{1}^{\left( 1 \right)}y_{2}^{\left( 1 \right)}- d_{-1}y_{3}^{\left( 1 \right)} \text{contribution to rate of change of VEGF+anti-VEGF complex concentration}$

**Clearance rates**

The concentration of VEGF, anti-VEGF and VEGF+anti-VEGF complexes in the vitreous are also modified by clearance rates. Within this compartment, we denote the vitreal clearance rates of VEGF, anti-VEGF and VEGF+anti-VEGF complexes by, $\kappa_{1}$, $\kappa_{2}$and $\kappa_{3}$, respectively. Direct measurement of clearance rates from the two compartments is difficult. However, if we assume that the clearance rate is inversely proportional to the size of the molecule then we can estimate the clearance rate for one molecular species from that of another. Following Stefanini et al.[^11^](#_ENREF_11) we use the relation proposed by Venturoli and Rippe[^12^](#_ENREF_12) to estimate the Stokes-Einstein radius of each molecule based upon its molecular weight, namely,

$$\text{Stokes-Einstein radius ≅ 0.483 ×}\left( \text{Molecular Weight} \right)^{0.386} \text{Eq.(S.5) }$$

Given that we are assuming a globular molecule, with $\text{Molecular Volume \textasciitilde}\left( \text{Stokes-Einstein radius} \right)^{3}$ we have to good approximation, $\text{Molecular Volume ∝ Molecular Weight}$, and thus clearance rates for other molecular species can be estimated from that for VEGF or anti-VEGF by taking the clearance rate to be inversely proportional to molecular weight. In this way we can obtain two estimates for the clearance rate of the VEGF+anti-VEGF complexes, and so we take the average, i.e. we set,

$$\kappa_{3}=\frac{1}{2} \left( \frac{{MW}_{\text{VEGF}}\kappa_{1}+ {MW}_{\text{anti-VEGF}}\kappa_{2}}{{{MW}_{\text{VEGF}}+ M}_{\text{anti-VEGF}}} \right) \text{Eq.(S.6)}$$

where ${MW}_{\text{VEGF}}$ and ${MW}_{\text{anti-VEGF}}$ are the molecular weights of VEGF and the anti-VEGF agent.

We set the molecular weight of VEGF to be 45kDa [^13^](#_ENREF_13) . From the FDA injection labels, we take anti-VEGF molecular weight to be 149kDa for bevacizumab and 115kDa for aflibercept.

To set a value for $\kappa_{2}$ we note that Park et al. [^14^](#_ENREF_14) quote a vitreal half-life for aflibercept in rabbit eyes of 3.92 days. This is smaller than the value of 7.1days derived by Stewart [^15^](#_ENREF_15) from a mathematical model (albeit more sophisticated than the estimation method encapsulated in Eq.(S.6)). Despite the estimates obtained by Park et al. being based upon a rabbit model, recent analysis by del Amo and Urtti [^16^](#_ENREF_16) suggests that pharmacokinetic parameter estimates obtained from rabbit models can be reliably translated (in absolute terms) to estimates for human eye pharmacokinetic parameters. Therefore, we take the human vitreal half-life of aflibercept to be 4 days. This then gives a value of $\kappa_{2}=0.173 \text{day}^{-1}$.

Setting a value for $\kappa_{1}$ is also problematic as experimentation with introduction of exogenous VEGF is rarely done. Instead we will again assume that clearance rates are inversely proportional to molecular weight and estimate $\kappa_{1}$ from the clearance rate of another anti-VEGF agent, in this case bevacizumab. We have chosen bevacizumab as our reference vitreal clearance rate as it appears to have a consistently estimated vitreal half-life of around 5-8 days (see Klettner 2014 [^17^](#_ENREF_17) for references). As bevacizumab is a 149 kDa molecule and therefore significantly larger than aflibercept, we take the upper end of this range for the vitreal half-life of bevacizumab. Consequently, we set,

$$\kappa_{1}= \frac{149}{{MW}_{\text{VEGF}}} \times\frac{1}{8}\times\ln2 \text{day}^{-1}=0.287 \text{day}^{-1} \text{Eq.(S.7) }$$

We use this value for $\kappa_{1}$ irrespective of which anti-VEGF agent we are modelling.

VEGF enters the first compartment due to clearance from the second compartment. Likewise, anti-VEGF and VEGF+anti-VEGF complexes can enter the first compartment due to clearance from the second compartment. For any molecular species we can simply express (to first approximation) the rate of clearance from the second compartment into the first in terms of its clearance rate from the first compartment, with adjustment for the relative sizes of the surface area, $S^{(c)}$, of the common region between the two compartments and the surface area, $S^{(1)}$, of the first compartment.

Thus the overall contributions to the rates of change within the first compartment due to clearance from and into the compartment are,

$$\frac{S^{(c)}}{S^{(1)}}{\kappa_{1}y}_{1}^{\left( 2 \right)}- \kappa_{1} y_{1}^{\left( 1 \right)} \text{ contribution to rate of change of VEGF concentration}$$

$${\frac{S^{(c)}}{S^{(1)}}\kappa_{2}y}_{2}^{\left( 2 \right)}-\kappa_{2} y_{2}^{\left( 1 \right)} \text{ contribution to rate of change of anti-VEGF concentration}$$

$${\frac{S^{(c)}}{S^{(1)}}\kappa_{3}y}_{3}^{\left( 2 \right)}-\kappa_{3} y_{3}^{\left( 1 \right)} \text{contribution to rate of change of VEGF+anti-VEGF complex concentration}$$

Putting together the contribution from production, binding, dissociation, and clearance from/to the compartment, we have the following differential equations that control the VEGF, anti-VEGF, and VEGF+anti-VEGF complex concentrations within the first compartment,

$$\frac{dy_{1}^{(1)}}{dt}= - d_{1}y_{1}^{\left( 1 \right)}y_{2}^{\left( 1 \right)}+ d_{-1}y_{3}^{\left( 1 \right)}+ \frac{S^{\left( c \right)}}{S^{\left( 1 \right)}}{\kappa_{1}y}_{1}^{\left( 2 \right)}- \kappa_{1} y_{1}^{\left( 1 \right)} \text{Eq.(S.8) }$$

$$\frac{dy_{2}^{(1)}}{dt}= f_{2}\left( t \right)- d_{1}y_{1}^{\left( 1 \right)}y_{2}^{\left( 1 \right)}+ d_{-1}y_{3}^{\left( 1 \right)}+ {\frac{S^{\left( c \right)}}{S^{\left( 1 \right)}}\kappa_{2}y}_{2}^{\left( 2 \right)}-\kappa_{2} y_{2}^{\left( 1 \right)} \text{Eq.(S.9)}$$

$$\frac{dy_{3}^{(1)}}{dt}= d_{1}y_{1}^{\left( 1 \right)}y_{2}^{\left( 1 \right)}- d_{-1}y_{3}^{\left( 1 \right)}+ {\frac{S^{\left( c \right)}}{S^{\left( 1 \right)}}\kappa_{3}y}_{3}^{\left( 2 \right)}-\kappa_{3} y_{3}^{\left( 1 \right)} \text{Eq.(S.10) }$$

The vitreous compartment we consider to be approximately spherical, of radius, $R=1\text{cm}$,[^18^](#_ENREF_18) and so its volume, $V^{(1)}$, and surface area, $S^{(1)}$, are easily calculated. For the second compartment, we approximate with a cuboid geometry with the upper and lowers surfaces being a square of size 25 disc areas, i.e. we set $S^{(c)}$ = 25 disc areas. We take the height, $H^{(2)}$, of the 2^nd^ compartment to be $0.5 \times\sqrt{\text{Disc Area}} (\cong0.78\text{mm}$ for a disc area of 2.4mm^2^). This gives the overall surface area of the second compartment as $S^{(2)}=60$ disc areas, and its volume, $V^{(2)}$, as $12.5\times\text{Disc Area}^{\frac{3}{2}}$.

## Compartment 2 (Retinal)

Within the second compartment the reaction kinetics controlling the evolution of the concentrations of VEGF, anti-VEGF, and VEGF+anti-VEGF complexes are the same as for compartment 1. In addition, VEGF also binds (and dissociates from) VEGF receptors on the endothelial cells of the existing choriocapillaris within the second compartment and on the new ingressed vasculature.

**Binding, Dissociation and Clearance of VEGF, anti-VEGF, VEGF+anti-VEGF complexes and VEGF receptors**

We denote by $y_{4}$ the proportion of VEGF receptors in the retinal compartment that are free (unbound), and likewise we use $y_{5}$ to denote the proportion of bound VEGF receptors in the retinal compartment. Clearly, $y_{4}+ y_{5}=1$. We use $\sigma_{R}$ to denote the VEGFR2 receptor density per unit area of choroid in the retinal compartment, and we use $C_{0}$ and $C_{CNV}$ to denote the surface area of the existing and ingressed choroid in the retinal compartment, respectively. Finally, $d_{2}$ and $d_{-2}$ represent, respectively, the rates for binding rate of VEGF to VEGF receptors, and for dissociation of VEGF from VEGF receptors. Following Stefanini et al.[^11^](#_ENREF_11) we set these equal to, $d_{2}= {10}^{7}\text{L}\text{Mol}^{-1}s^{-1}$ and $d_{-2}= {10}^{-3}\text{s}^{-1}$.

Consequently, the evolution equations for the retinal compartment are,

$$\frac{dy_{1}^{\left( 2 \right)}}{dt}= f_{1}\left( t \right)- d_{1}y_{1}^{\left( 2 \right)}y_{2}^{\left( 2 \right)}+ d_{-1}y_{3}^{\left( 2 \right)}- d_{2}{\left( \sigma_{R}C_{0} + \sigma_{R}C_{CNV} \right)y}_{1}^{\left( 2 \right)}y_{4}+ d_{-2}\left( \sigma_{R}C_{0} + \sigma_{R}C_{CNV} \right)y_{5}$$

$$+ {\frac{S^{\left( c \right)}}{S^{\left( 1 \right)}}\frac{V^{\left( 1 \right)}}{V^{\left( 2 \right)}}\kappa_{1}y}_{1}^{\left( 1 \right)}-\frac{S^{\left( 2 \right)}}{S^{\left( 1 \right)}}\frac{V^{\left( 1 \right)}}{V^{\left( 2 \right)}}\kappa_{1}y_{1}^{\left( 2 \right)} \text{Eq.(S.11) }$$

$$\frac{dy_{2}^{(2)}}{dt}= - d_{1}y_{1}^{\left( 2 \right)}y_{2}^{\left( 2 \right)}+ d_{-1}y_{3}^{\left( 2 \right)} + {\frac{S^{\left( c \right)}}{S^{\left( 1 \right)}}\frac{V^{\left( 1 \right)}}{V^{\left( 2 \right)}}\kappa_{2}y}_{2}^{\left( 1 \right)}-\frac{S^{\left( 2 \right)}}{S^{\left( 1 \right)}}\frac{V^{\left( 1 \right)}}{V^{\left( 2 \right)}}\kappa_{2} y_{2}^{\left( 2 \right)} \text{Eq.(S.12)}$$

$$\frac{dy_{3}^{(2)}}{dt}= d_{1}y_{1}^{\left( 2 \right)}y_{2}^{\left( 2 \right)}- d_{-1}y_{3}^{\left( 2 \right)}+{\frac{S^{\left( c \right)}}{S^{\left( 1 \right)}}\frac{V^{\left( 1 \right)}}{V^{\left( 2 \right)}}\kappa_{3}y}_{3}^{\left( 1 \right)}- \frac{S^{\left( 2 \right)}}{S^{\left( 1 \right)}}\frac{V^{\left( 1 \right)}}{V^{\left( 2 \right)}} \kappa_{3}y_{3}^{\left( 2 \right)} \text{Eq.(S.13) }$$

# A model that relates the levels of VEGF and anti-VEGF to level of bound VEGF receptors.

A key aspect of our model is to determine the effects of VEGF. This is represented by the fraction of VEGF receptors that are bound to VEGF at any time. This is turn requires an understanding of the levels of available VEGF and of available receptor domains. Evolution of the concentrations of VEGF, anti-VEGF, and VEGF+anti-VEGF complexes are modelled in the preceding sections. We now consider the modelling of the available receptor domains.

## VEGF receptor density $\boldsymbol{\sigma}_{\boldsymbol{R}}$

We consider the density of VEGF receptors (per unit area of choroid) to be constant, i.e. is the same over any reasonably large area of the choriocapillaris and the ingressed choroid. We denote this density by $\sigma_{R}$.

VEGF receptor densities for choriocapillaris endothelial cells are not known. VEGF receptor types are either VEGFR1 or VEGFR2. VEGFR2 is the pro angio-genic receptor, and so we focus upon the density of VEGFR2. Previous studies have reported values of 6000-150000 VEGFR2 per cell, though according to Imoukhede and Popel[^19^](#_ENREF_19) this large variation was attributed to the use of non-human, clonal and transfected cells. A more reliable figure would be that of 12400 VEGFR2 per cell obtained by Chen et al.[^20^](#_ENREF_20) for human umbilical vein endothelial cells. More recently, Imoukhede and Popel[^19^](#_ENREF_19) obtained in vitro estimates of 5800 ± 300 VEGFR2 per cell from human umbilical vein endothelial cell, human dermal microvascular endothelial cells, and human dermal lymphatic microvascular endothelial cells. However, Imoukheude and Popel note that estimates of VEGFR2 densities are typically higher in vitro than in vivo. Consequently, for human choriocapillaris endothelial cells we use a density of 5000 VEGFR2 per cell.

Endothelial cells are typically considered to be relatively thin hexagonal shaped cells. Endothelial cell sizes vary naturally depending on the function of the corresponding vasculature. Older sources quote estimates of endothelial cell lengths of up to 30 $\mu$m,[^21^](#_ENREF_21) whilst more recent studies estimate cell areas of 245 $\mu$m^2^ and 71 $\mu$m^2^ for human common iliac and hepatic arteries, respectively.[^22^](#_ENREF_22) As common iliac arteries represent larger vessels, we would naturally expect choroid capillary endothelial cells to be closer in area to those from the hepatic artery, or indeed other small vessels. Human umbilical vein endothelial cells are quoted as having a length in the range 14-15$\mu$m.[^23^](#_ENREF_23) From this it seems reasonable to take choriocapillaris endothelial cells as having an area in the range 50-100 $\mu$m^2^ , and for our simulations we have chosen to take the upper end of this range. Combining this value with the number of VEGFR2 receptors per cell, we arrive at a value of,

$$\sigma_{R}=5 \times{10}^{13}\text{ VEGFR2 }\text{m}^{-2} \text{Eq.(S.14) }$$

## The rate of change in the number of bound VEGF receptors

The total choroid area in the second compartment is $C_{0}+ C_{CNV}$, where $C_{0}$ is the choroid area within in the retinal compartment of the existing choriocapillaris, and $C_{CNV}$ is the choroid area of the new ingressed vasculature. Consequently, the number of bound VEGF receptors at any point in time is given by $\sigma_{R}(C_{0}+ C_{CNV})y_{5}$, whilst the number of free VEGF receptors at any point in time is given by $\sigma_{R}(C_{0}+ C_{CNV})y_{4}$ .

The rate of change in the number of bound VEGF receptors is determined by the number of free receptors, the free VEGF concentration and the dissociation rate of the existing bound VEGF receptors. Although the ingressed choroid is not continuously moving within the sub-retinal edema, we do consider it to be, to first approximation, uniformly dispersed. Consequently, with the VEGF molecules being mobile within the second compartment we still apply standard second order reaction kinetics to obtain,

$$\frac{d\sigma_{R}(C_{0}+ C_{CNV})y_{5}}{dt}= d_{2}\sigma_{R}\left( C_{0}+ C_{CNV} \right){y_{4}y}_{1}^{\left( 2 \right)}+ d_{-2}\sigma_{R}(C_{0}+C_{CNV})y_{5}$$

$${\Rightarrow y}_{5}\frac{d(C_{0}+ C_{CNV})}{dt} + (C_{0}+ C_{CNV})\frac{dy_{5}}{dt}= d_{2}\left( C_{0}+ C_{CNV} \right)(1- {y_{5})y}_{1}^{\left( 2 \right)}+ d_{-2}(C_{0}+C_{CNV})y_{5}$$

$$\Longrightarrow\frac{dy_{5}}{dt}= d_{2}(1- {y_{5})y}_{1}^{\left( 2 \right)}+ d_{-2}y_{5} - y_{5}\frac{d\log\left( C_{0}+ C_{CNV} \right)}{dt} \text{Eq.(S.15) }$$

Evaluation of $C_{0}$ is more difficult. We consider an idealized cuboid geometry for the second compartment, of linear dimensions $x_{1}= \text{Width = }\sqrt{S^{(c)}}$, $x_{2}= \text{Height = }H^{(2)}$, $x_{3}= \text{Depth = }\sqrt{S^{(c)}}$, and we denote the typical capillary radius within the existing choriocapillaris denoted by $R_{C}$. Then, if the and typical cross-sectional packing fraction of the capillaries within the choriocapillaris is denoted by $PF$, we have to first approximation,

$$\frac{x_{1}x_{2}PF}{\pi R_{C}^{2}}= \frac{C_{0}}{x_{3}2\pi R_{C}} \Longrightarrow C_{0}= \frac{2PF \times x_{1}x_{2}x_{3}}{R_{C}} = \frac{2PF V^{\left( 2 \right)}}{R_{C}} \text{Eq.(S.16) }$$

The cross-sectional packing fraction is the typical proportion of a planar cross-section of the 2^nd^ compartment that is occupied by cross-sections of choroid vessels. We set $PF=0.4$. This value for the packing fraction is based upon assessment of Fourier-domain optical coherence tomography images. For the typical radius of a choroid capillary in the 2^nd^ compartment we take$R_{C}=40\mu\text{m}$. This value is taken from Staurenghi et al.,[^24^](#_ENREF_24) who give the average radius of feeder vessels from the choriocapillaris to the sub-retinal region to be $40.5\mu\text{m}$.

# A model that relates the level of bound VEGF receptors to the rate of growth of ingressed choroid.

We have modelled above the interactions of VEGF, anti-VEGF and receptors including development of functions that describe levels of bound VEGF. We examine now exactly how this bound VEGF leads to abnormal choroidal growth.

We acknowledge that the binding of VEGF to VEGF receptors is more complex than we have currently alluded to. VEGF is in fact a family of VEGF proteins (VEGF-A, VEGF-B, VEGF-C, VEGF-D and Placental Growth Factor), with VEGF-A being the pro-angiogenic factor of these five.[^13^](#_ENREF_13)^,^ [^25^](#_ENREF_25)^,^ [^26^](#_ENREF_26) VEGF-A itself comes in several isoforms (differentially denoted by molecular weight). Of the various isoforms VEGFA_165_ is the most abundant, though VEGFA_121_ and VEGF_189_ are also common.[^26^](#_ENREF_26) VEGF proteins bind to a number of receptor tyrosine kinases, VEGFR1, VEGFR2, and VEGFR2. VEGFA binds predominantly as a homo-dimer to VEGFR1 and VEGFR2, with binding to VEGFR2 being considered the most important signal transduction process for angiogenesis.[^25^](#_ENREF_25)^,^ [^26^](#_ENREF_26) Autophosphorylation of internal domains of the receptors can then activate multiple signalling pathways and downstream processes.[^25^](#_ENREF_25)

Given the plethora of VEGF proteins, of various isoforms and variety of receptors, modelling the full biochemical detail of this signalling cascade is complicated. Indeed, VEGF mediated tumour angiogenesis models[^8^](#_ENREF_8) can involve up to 38 separate differential equations to describe the evolution of the various molecular species concentrations – even before the physiological response to those changing concentrations has been modelled. Consequently, we pragmatically use a simplified model for the pro-angiogenic effect of bound VEGF receptors. Specifically, we develop a model that considers a single VEGF form and a single VEGF receptor form. Therefore, the parameters we use for modelling the single VEGF form will be those appropriate (predominantly) to VEGFA_165_. Likewise, the parameters we use for modelling the single VEGF receptor form will be those appropriate to VEGFR2.

However, even with this simplification, modelling the precise mechanism by which raised bound VEGF receptor levels lead to increased growth of the ingressed choroid is complex, as the downstream mechanism linking this signalling cascade to the neovascularization process has not been fully elucidated. Therefore, we propose a heuristic model for the choroid growth, whereby the rate of growth of $C_{CNV}$ is simply proportional to the total signal transduced by bound VEGF receptors. As a background level of VEGF is required even to maintain the choriocapillaris under normal conditions,[^26^](#_ENREF_26)^,^ [^27^](#_ENREF_27) we actually make the rate of growth of $C_{CNV}$ only proportional to the transduced signal above a specified threshold. So the simplest model we can propose takes the form,

$$\frac{dC_{CNV}}{dt}= a_{1}\sigma_{R}\left( b_{0}C_{0}+ C_{CNV} \right) \left( \frac{y_{5}}{a_{2}}-1 \right) \text{Eq.(S.17) }$$

The parameter $a_{1}$determines the rate of choroid growth for a unit level of stimulus (as determined by $\sigma_{R}(b_{0}C_{0}+ C_{CNV})$).

The parameter $a_{2}$ determines when the level of bound VEGF-receptors will result in further choroid growth and when the level of bound VEGF-receptors is insufficient to maintain the current level of ingress. The value $a_{2}$ represents the bound VEGF-receptor fraction needed to maintain the current ingress. Under normal conditions and during embryonic developmental stages neovascularization is inhibited within the region above the choriocapillaris by factors such as TIMP-3 and PEDF that are expressed by RPE cells and localized to the RPE.[^28^](#_ENREF_28)^,^ [^29^](#_ENREF_29) TIMP-3 and PEDF restrict angiogenesis via binding to VEGFR2 (in competition with VEGF),[^30^](#_ENREF_30)^,^ [^31^](#_ENREF_31) albeit with much weaker interaction than VEGF. When CNV occurs and choroid ingresses above the RPE, the presence of TIMP-3 and PEDF will result in competition for VEGFR2, and therefore a lower level of receptors available for VEGF stimulated angiogenesis. Due to the weaker level of association (compared to VEGF) of TIMP-3 and PEDF with VEGFR2, the fraction of VEGFR2 bound by TIMP-3 and PEDF will change on slower timescales than the changes in VEGF bound VEGFR2. Consequently, when modelling the kinetics of VEGF-VEGFR2 binding we consider the fraction of TIMP-3 and PEDF bound VEGFR2 to be quasi-stationary. Overall we consider the effect of TIMP-3 and PEDF to simply be a reduction in the effective density of free receptors, and consequently an increase in the effective value of $a_{2}$ . We model this by taking the effective value of $a_{2}$ to be dynamic, and a simple increasing function of edema size $S_{edema}$. The simple functional form we use for $a_{2}$ is given in Eq.(18) below.

$$a_{2}\left( S_{edema} \right)= \frac{a_{2}^{(0)}\text{exp}\left( {S_{edema}}/{S_{edema}^{(ref)}} \right)}{1+ a_{2}^{(0)}\left( \text{exp}\left( {S_{edema}}/{S_{edema}^{(ref)}} \right)-1 \right)} \text{Eq.(S.18) }$$

We have made the value of $a_{2}$ progressively increase with increasing edema size to reflect the fact that, as VEGF and CNV levels increase, we expect to see an upregulation of PEDF.[^32^](#_ENREF_32)^,^ [^33^](#_ENREF_33) Therefore, with increasing edema size we expect to see an increasing fraction of VEGFR2 receptors to be bound by PEDF and TIMP-3. We set $a_{2}^{(0)}$ equal to the steady state bound fraction of VEGF receptors reached when there is no CNV, and when VEGF production in the second compartment is held constant at a rate required to achieve the observed clinical concentration of VEGF in the first compartment for unaffected patients. The reference edema size, $S_{edema}^{(ref)}$, determines how rapidly the fraction of VEGFR2 receptors not bound by TIMP-3 or PEDF decreases with increasing edema growth. We set $S_{edema}^{(ref)}$ to be 9 optic disc areas, so that a significant increase in $a_{2}$ (from approximately 4.7% to approximately 11.7%) occurs even when $S_{edema}$ has only reached about $\frac{1}{2}$ of the largest values we have observed in a clinical sample (excluding outliers - see Section 5 for details ).

The parameter $b_{0}$ is between 0 and 1, and represents the fact that although VEGF receptors will be bound throughout the entire choroid area within the second compartment, only a small fraction of $C_{0}$ will be capable of contributing to the new growth – namely only that portion of the existing choroid that is capable of breaking through the Bruch’s membrane and RPE into the sub-retinal space. This will be where the Bruch’s membrane and RPE have been sufficiently weakened, e.g. due to the presence of Drusen. Therefore, we regard $b_{0}$ to represent the probability that an existing area of choriocapillaris will break through into the sub-retinal space and so $b_{0}$ will be determined by the density of weakened areas of the Bruch’s membrane and RPE. We have set $b_{0}=0.1$, i.e. 10% of the Bruch’s membrane within the second compartment is considered at risk of being broken through by the new vasculature. This number is has been chosen by clinical judgement due to absence of experimental evidence.

Finally to set a value for the parameter $a_{1}$ we note that Vander et al.[^34^](#_ENREF_34) give a mean ingressed choroid growth rate of $18\mu\text{m per day}$ for AMD affected patients. Consequently, we set a target growth rate, $C_{CNV}^{(Target)}=2\pi R_{C}\times18\mu\text{m }\text{day}^{-1}=7.2\times{10}^{-4} \text{mm}^{2} \text{day}^{-1}$ for the ingressed choroid area. Setting $a_{1}\sigma_{R}b_{0}C_{0}=C_{CNV}^{(Target)}$will ensure that $a_{1}$ is set to achieve appropriate growth rates for $C_{CNV}$. During the growth of the edema $C_{CNV}$ may become significantly larger than $b_{0}C_{0}$, but equally we will have $y_{5}- a_{2}<1$, and so we regard a value of $2\pi R_{C}\times18\mu\text{m per day}$ for $a_{1}\sigma_{R}b_{0}C_{0}$ to be a not unreasonable figure to use across the full range of observed edema sizes. With this choice we checked that the maximum height achieved for an untreated edema is in line with the largest values observed clinically.

# A model that relates the amount of ingressed choroid to the rate of growth of the edema.

We now turn our attention to the last stage of our modelling, describing a model that relates the amount of ingressed choroid to the consequent rate of growth of the edema. Ultimately, it is the edema volume$V_{edema}$, and its change over time and in response to injection of anti-VEGF agents that we are interested in modelling. More likely, it is the edema base area $S_{edema}$ and edema height that are clinically measurable, so it is these quantities that we actually focus upon.

Growth of the edema is due to increased amounts of fluid leaking from the newly stimulated ingressed choroid. To model the edema growth we must now model the dynamics of the fluid leakage from this ingressed choroid into the edema and clearance of this fluid from the edema.

We assume the rate of fluid leakage from the ingressed choroid is proportional to the surface area of the ingress, i.e., that the density of pores through which leakage occurs is approximately constant as the ingress grows or contracts.

Fluid that leaks or is exuded from the ingressed choroid is ultimately cleared through channels within the RPE layer. In a similar fashion, we assume the density of channels in the RPE through which fluid clearance can occur is approximately constant, and therefore the rate of clearance is proportional to the base area of the edema,$S_{edema}$. Thus fluid enters the edema at a rate $\sigma_{c}r_{c}C_{CNV}$, where $\sigma_{c}$ is the pore density on the ingressed choroid, $r_{c}$ is the fluid exudation rate from a single pore on the ingressed choroid, and $C_{CNV}$ is the surface area of the ingressed choroid. Similarly, fluid exits the edema at a rate $\sigma_{edema}r_{edema}S_{edema}$ , where $\sigma_{edema}$ is the pore density in the base of the edema and $r_{edema}$ is the fluid exudation rate from a single pore on the base of the edema. We assume that the fluid within the edema is incompressible, so that growth in the edema volume is simply governed by the difference in the two exudation rates described above. That is, the evolution of the edema volume, $V_{edema}$, is described by,

$\frac{dV_{edema}}{dt}= \sigma_{C}r_{c}C_{CNV}- \sigma_{edema}r_{edema}S_{edema} \text{Eq.(S.19) }$

For the edema geometry we assume that we have radial symmetry, i.e., the edema can be described as the cap of a sphere. In cross section the edema has geometry as shown in Supplementary Figure 1 below,


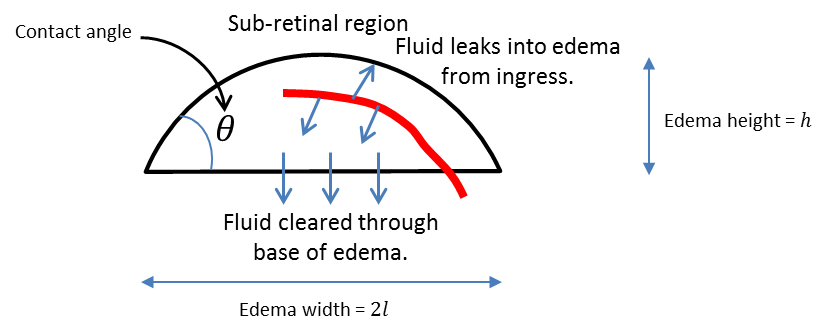


Supplementary Figure 1: Schematic of the main processes controlling the fluid volume within the edema.

The dimensions of the edema and the contact angle $\theta$ are related via,

$$l=h \sqrt{\frac{\left( 1+\cos\theta\right)}{\left( 1-\cos\theta\right)}} \text{Eq.(S.20) }$$

In our idealized model we regard the thickness of the interface between the edema and the vitreous to be very much smaller than the edema itself, and so the contact angle $\theta$ will most likely be a function only of the retina properties, i.e. its elastic characteristics resulting from the strength of integrin/adherin binding between retinal cells and between retinal cells and the RPE. Thus we expect $\theta$ to be roughly constant, particularly as the edema becomes larger. With a constant contact angle $\theta$ simple trigonometry gives the following relationship between edema volume, $V_{edema}$, and edema base area, $S_{edema}$ ,

$V_{edema}= {\left[ \frac{\left( \frac{2}{3}- \cos\theta+ \frac{1}{3}\text{cos}^{3}\theta\right)}{\sqrt{\pi}\left( 1-\text{cos}^{2}\theta\right)^{\frac{3}{2}}} \right] \times S}_{edema}^{\frac{3}{2}}= x\left( \theta\right) \times S_{edema}^{\frac{3}{2}} \text{Eq.(S.21) }$

and so we have an growth equation for the edema base area,

$x\frac{dS_{edema}^{3/2}}{dt}= \sigma_{C}r_{c}C_{CNV}- \sigma_{edema}r_{edema}S_{edema} \text{Eq.(S.22) }$

Despite other model geometries within the existing literature assuming an incident angle of 90°, we expect the contact angle$\theta$ to be less than 90°. Simple physics shows that if the contact angle is not less than 90° then equilibrium cannot be reached for the lateral surface tension forces acting at the junction between the RPE and the retina, and separation of the RPE and retina would continue indefinitely.

To set a suitable value for the contact angle we have analysed measurements of edema height and edema width from a sample of 41 patients. The distributions of observed edema heights and edema widths are shown in the two histograms below.


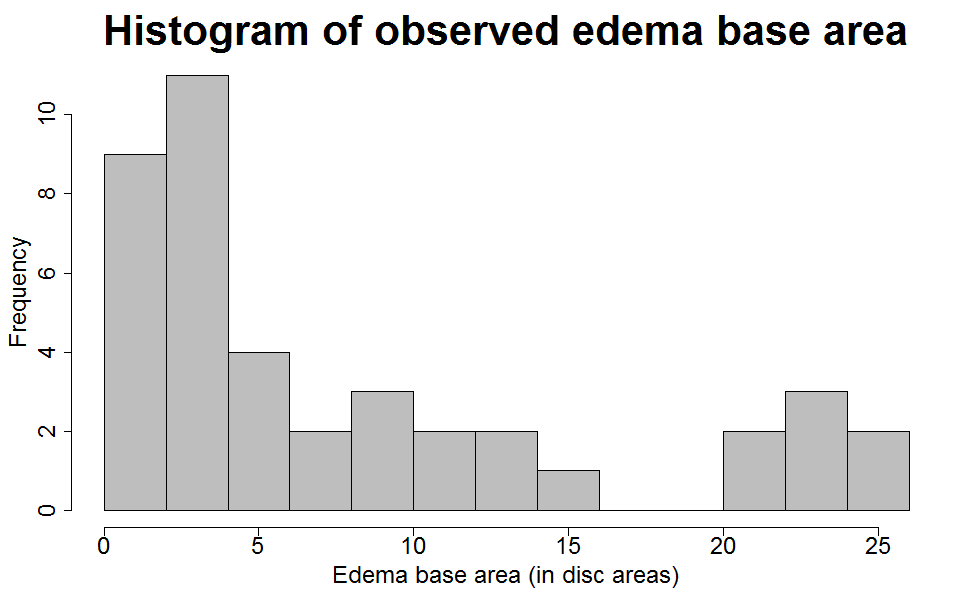

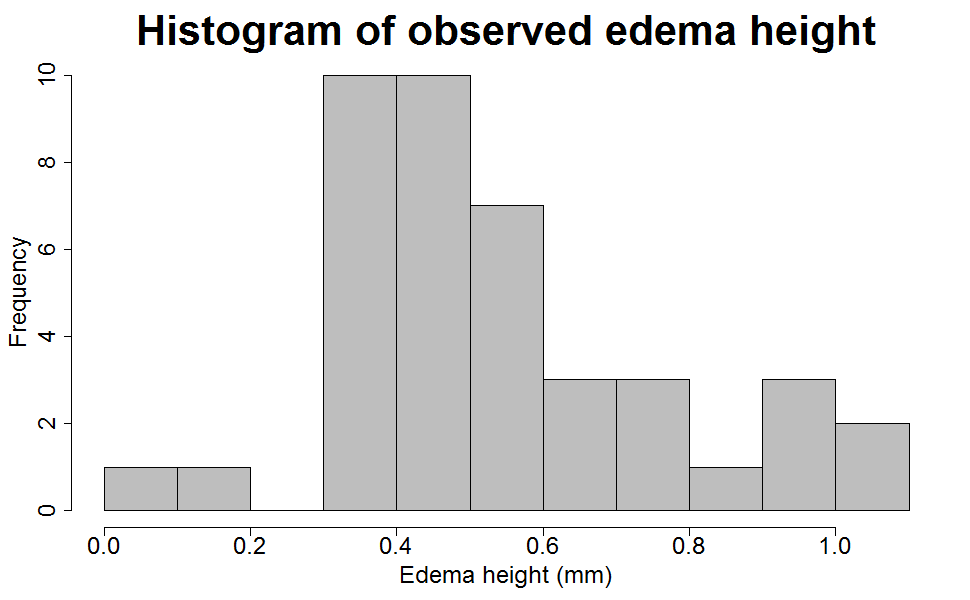


Supplementary Figure 2: Distributions of edema base area and edema height from a sample of 41 patients.

For each patient we can convert the measured edema height and width into an estimate of the patient specific contact angle, using Eq.(S.20). The distribution of patient specific contact angles is shown in the histogram in Supplementary Figure 3 below. Despite a wide range of observed edema base area, we see a strong peak in the distribution of the contact angles around the modal value, confirming that the contact angle is largely a property of the microscopic characteristics of the system, with patient-to-patient variability superimposed. Indeed, the right hand graph of Supplementary Figure 3 below shows the variation of contact angle estimates with edema size (edema base area) across the 41 sample points, along with the (loess) trend line. As hypothesized, the trend in the contact angle tends to a level value as the edemas become bigger – in this case above approximately 5 (reference) disc areas. The mean and median of the patient specific contact angles are 32.9°, and 31.4°, respectively. Consequently, for the contact angle, we set $\theta=30^{\circ}$.


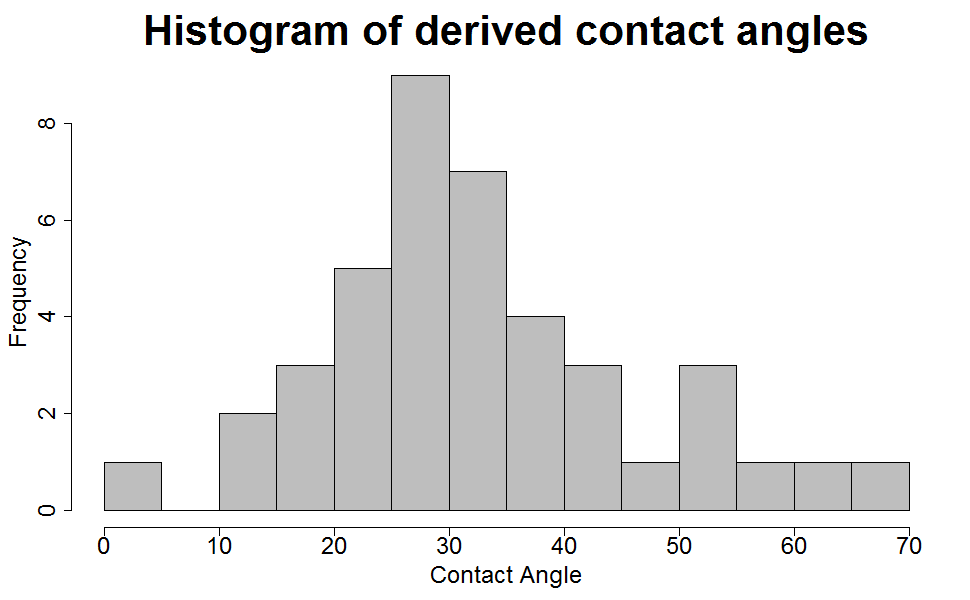

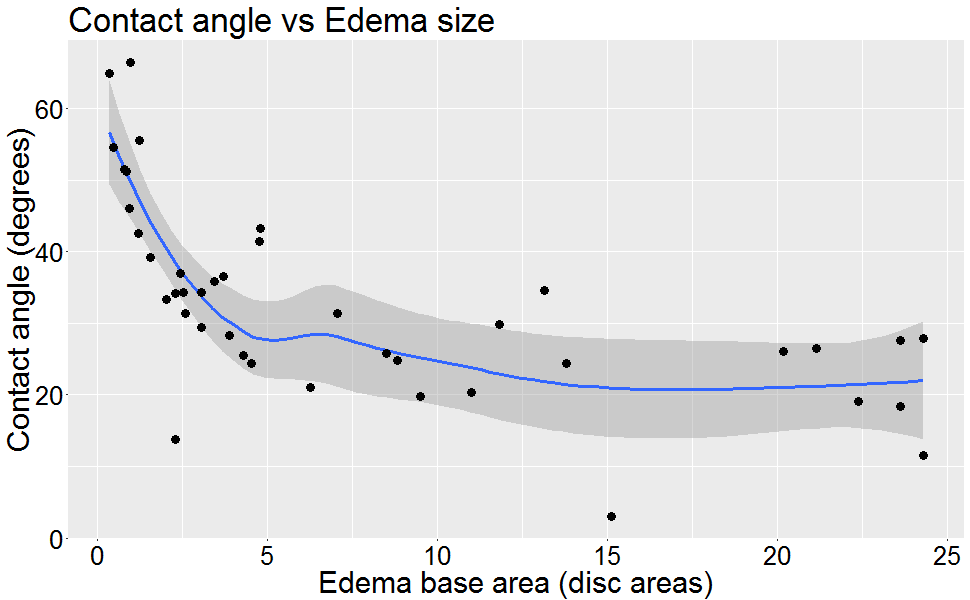


Supplementary Figure 3: Distribution and trend of patient-specific contact angle estimates.

Returning to the edema size evolution equation we note that if $\sigma_{edema}r_{edema}$ were relatively large, then the edema volume, and hence edema base area, would equilibrate quickly for a fixed value of$C_{CNV}$ . If $\sigma_{edema}r_{edema}$ was sufficiently large that equilibration of $S_{edema}$ were on a faster timescale than the evolution of $C_{CNV}$, then we would have a quasi-equilibrium between $S_{edema}$ and $C_{CNV}$ at all time points, i.e.,

$S_{edema}\left( t \right)= \frac{\sigma_{C}r_{C}}{\sigma_{edema}r_{edema}}C_{CNV}\left( t \right) \text{Eq.(S.23) }$

We use Eq.(S.23) to help determine a suitable value for $\sigma_{C}r_{C}$. Stamer et al.[^35^](#_ENREF_35) give the typical fluid clearance rate as $\text{3-4 }\mu\text{L}\text{cm}^{-2}\text{h}^{-1}$. Consequently, we set $\sigma_{edema}r_{edema}=4\mu\text{L}\text{cm}^{-2}\text{h}^{-1}$ .To set a value for $\sigma_{C}r_{C}$ we use the equilibrium relationship between $S_{edema}(t)$ and $C_{CNV}(t)$ given in Eq.(S.23) and note that Vander et al.[^34^](#_ENREF_34) give an ingressed choroid growth rate of $18\mu\text{m per day}$. Staurenghi et al.[^24^](#_ENREF_24) give the average radius of feeder vessels from the choriocapillaris to the sub-retinal region to be $40.5\mu\text{m}$, and so we assume an ingressed choroid capillary radius of $R_{C}=40\mu\text{m}$, giving a typical growth rate for $C_{CNV}$ of $C_{CNV}^{(Target)}= 7.2\times{10}^{-4}\text{mm}^{2}$ per day. From the distribution of observed edema base areas in our sample of patients we take the maximum edema base area achieved (prior to treatment) to be of the order of 15 to 20 (reference) disc areas. Therefore we set a target value of $S_{edema}^{(Target)}=7.5 \text{Disc areas per year}$, for the untreated edema growth rate, so that maximum untreated edema would occur around 2 years after onset. With the reference disc area having been set to $2.4\text{mm}^{2}$ and using Eq.(S.23) we derive an estimate,

$$\frac{\sigma_{C}r_{C}}{\sigma_{edema}r_{edema}}\cong\frac{S_{edema}^{(Target)}}{C_{CNV}^{(Target)}}\cong10.9 . \text{Eq.(S.24)}$$

Consequently, we set $\sigma_{c}r_{c}=10.9 \times\sigma_{edema}r_{edema}=10.9 \times4 \mu\text{L}\text{cm}^{-2}\text{h}^{-1}$.

We emphasize that it is the differential equation Eq.(S.22) that we use for the evolution of the bleb size, not the equilibrium relationship in Eq.(S.23). We use the equilibrium relationship simply as a means for setting a suitable value for $\sigma_{c}r_{c}$.

# Statistical analysis and numerical computation

All statistical analysis and numerical computations were performed using the R statistical computing language.[^36^](#_ENREF_36) The set of coupled differential equations that represent the various component models we have outlined above were solved numerically using the deSolve package for R.[^37^](#_ENREF_37) Numerical solution was done piecewise between injection time-points. The step-size for numerical integration of the differential equations was ensured to be sufficiently small. Typically we set the step-size so that we had several thousand time-steps per month of chronological time. We checked that this resulted in a sufficiently small enough step size by observing that no discernible change in numerical output was observed when we further halved the step-size.

# Variables and Parameter values

Below we recap the main variables and parameters used in our modelling. Table 1 below gives the variables that we calculate using the differential equations outlined in the preceding sections. Table 2 gives the values or derivations of the parameters for all parameters introduced in the text of the Supporting Information. Note that some parameters are full model parameters, in that they appear explicitly within the differential equations, whilst others are merely used to set values for the model parameters. In Table 2 we specifically give the parameter values used to produce the figures shown within the main text.

Table 1: Model variables - definitions

| **Variable** | **Definition** |
| --- | --- |
| $y_{1}^{(1)}$ | Concentration of VEGF in 1^st^ compartment |
| $y_{2}^{(1)}$ | Concentration of anti-VEGF in 1^st^ compartment |
| $y_{3}^{(1)}$ | Concentration of VEGF+anti-VEGF complexes in 1^st^ compartment |
| $y_{1}^{(2)}$ | Concentration of VEGF in 2^nd^ compartment |
| $y_{2}^{(2)}$ | Concentration of anti-VEGF in 2^nd^ compartment |
| $y_{3}^{(2)}$ | Concentration of VEGF+anti-VEGF complexes in 2^nd^ compartment |
| $y_{5}$ | Fraction of VEGFR2 receptors in the 2^nd^ compartment that are bound |
| $C_{CNV}$ | Surface area of ingressed neovascularization |
| $S_{edema}$ | Base area of the edema |

Table 2: Parameters - definitions and chosen values

| **Parameter** | **Definition** | **Derivation or Source** | **Value** |
| --- | --- | --- | --- |
| $v_{I}$ | Mass of anti-VEGF agent introduced into 1^st^ compartment due to a single injection. | From literature - Schmidt-Erfurth et al. (2014) | 2mg |
| $\omega_{0}$ | Typical vitreal concentration of VEGF in unaffected patients. | From literature – see Section 1.2, p4 | $10\text{ pg }\text{ml}^{-1}$ |
| $\omega_{1}$ | Typical vitreal concentration of VEGF in affected patients. | From literature – see Section 1.2, p4 | $100\text{ pg }\text{ml}^{-1}$ |
| $\tau_{1}$ | Reciprocal of rate at which healthy RPE cells become ischemic and move to an elevated rate of VEGF production. | Set using clinical judgement – see Section 1.2, p3 | 6 months |
| $\tau_{2}$ | Reciprocal of rate at which ischemic RPE cells cease elevated VEGF production. | Set using clinical judgement – see Section 1.2, p3 | 12 months |
| $\tau_{lag}$ | Lag between elevated VEGF production and initiation of neovascularization. | Set using clinical judgement – see Section 1.2, p3 | 1 month |
| $\phi$ | Ratio of post-ischemic RPE cell VEGF production rate to pre-ischemic RPE cell VEGF production rate. | Set using clinical judgement – see Section 1.2, p2 | 0 |
| $v_{0}N_{RPE}(0)$ | Rate of VEGF production within 2^nd^ compartment in unaffected patients. | Derived by computationally matching to a vitreal VEGF concentration of $\omega_{0}$ for unaffected patients – see Section 1.2, p3 | $7.04\times{10}^{-6} \text{ng }\text{ml}^{-1}\text{s}^{-1}$ |
| $v_{1}N_{RPE}(0)$ | Rate of VEGF production within 2^nd^ compartment in affected patients. | Derived by computationally matching a vitreal concentration of $\omega_{1}$ for AMD affected patients – see Section 1.2, p3 | $2.05\times{10}^{-4} \text{ng }\text{ml}^{-1}\text{s}^{-1}$ |
| $M_{\text{VEGF}}$ | Molecular weight of VEGF. | From literature – Ferrara et al. (2003) | 45kDa |
| $M_{\text{anti-VEGF}}$ | Molecular weight of anti-VEGF. | From FDA injection label | 115kDa |
| $M_{\text{VEGF+anti-VEGF}}$ | Molecular weight of VEGF+anti-VEGF complex. | $M_{\text{VEGF}}+ M_{\text{anti-VEGF}}$ | 160kDa |
| $\kappa_{1}$ | Clearance rate of VEGF from first compartment. | Derived from multiple literature sources and using Eq.(S.7) – see Section 2.1, p7 | $0.287 \text{day}^{-1}$ |
| $\kappa_{2}$ | Clearance rate of anti-VEGF from first compartment. | From multiple literature sources – see Section 2.1, p7 | $0.173 \text{day}^{-1}$ |
| $\kappa_{3}$ | Clearance rate of VEGF-anti-VEGF complexes from first compartment. | Derived from $\kappa_{2}$, ${MW}_{\text{VEGF}}$, and ${MW}_{\text{VEGF-anti-VEGF}}$ – see Eq.(S.6), Section 2.1, p6 | $0.103 \text{day}^{-1}$ |
| $d_{1}$ | Binding constant for binding of VEGF and anti-VEGF. | From multiple literature sources – see Section 2.1, p5 | ${10\times10}^{5}\text{L}\text{Mol}^{-1}\text{s}^{-1}$ |
| $d_{-1}$ | Dissociation constant for unbinding of VEGF from anti-VEGF. | From multiple literature sources – see Section 2.1, p5 | ${1\times10}^{-5}\text{s}^{-1}$ |
| $d_{2}$ | Binding constant for binding of VEGF and VEGF receptors. | From literature – Stefanini et al. (2008) – see Section 2.2, p9 | ${10}^{7}\text{L}\text{Mol}^{-1}\text{s}^{-1}$ |
| $d_{-2}$ | Dissociation constant for unbinding of VEGF from VEGF receptors. | From literature – Stefanini et al. (2008) – see Section 2.2, p9 | ${10}^{-3}\text{ }\text{s}^{-1}$ |
| $R$ | Radius of vitreous. | From literature – Tripathi et al. (1997) | 1cm |
| $S_{Disc}$ | Area of optic disc. | From literature – Quigley et al. (1990) | $2.4 \text{mm}^{2}$ |
| $S^{(1)}$ | Total surface area of first compartment. | Derived assuming spherical vitreal compartment and vitreal radius of 1cm - see Section 2.1, p8 | $4\pi\times100 \text{mm}^{2}$ |
| $V^{(1)}$ | Total volume of first compartment. | Derived assuming spherical vitreal compartment and vitreal radius of 1cm - see Section 2.1, p8 | $\frac{4}{3}\pi\times1000 \text{mm}^{3}$ |
| $S^{(c)}$ | Surface area of interface between first and second compartments. | 25 optic disc areas - see Section 2.1, p8 | 60mm^2^ |
| $H^{(2)}$ | Height of second compartment. | $0.5 \times\sqrt{\text{Disc Area}}$ - see Section 2.1, p9 | 0.78mm |
| $S^{(2)}$ | Total surface area of second compartment. | $2S^{(c)}+4H^{(2)}\sqrt{S^{(c)}}$ - see Section 2.1, p9 | 144mm^2^ |
| $V^{(2)}$ | Total volume of second compartment. | $S^{(c)}\times H^{(2)}$ - see Section 2.1, p9 | 46.5mm^3^ |
| PF | Cross-sectional packing fraction of choriocapillaris capillaries within the second compartment. | Eq.(S.16), Section 3.2, p12 | 0.4 |
| $R_{C}$ | Typical capillary radius within the 2^nd^ compartment. | From literature - Staurenghi et al. (1998) | $40\mu\text{m}$ |
| $C_{0}$ | Surface area of choriocapillaris within second compartment prior to neovascularization. | Derived from $PF$, $R_{C}$, and $V^{(2)}$ – see Eq.(S.16), Section 3.2, p12 | $930\text{mm}^{2}$ |
| $\sigma_{R}$ | Density (per unit area) of VEGFR2 receptors on the surface of choroid endothelial cells. | From literature – see Eq.(S.14), Section 3.1, p11 | $5\times{10}^{13}\text{m}^{-2}$ |
| $b_{0}$ | Proportion of capillary surface area in choriocapillaris that acts as potential source of CNV ingress into sub-retinal space. | Set using clinical judgement – see Section 4, p16 | 0.1 |
| $C_{CNV}^{\left( Target \right)}$ | Target CNV ingress growth rate. | From literature – Vander et al. (1989) - see Section 4, p16 | $2\pi R_{C}\times18\text{μm }\text{day}^{-1}$ |
| $a_{1}$ | Rate of increase in neovascularization surface area per unit of transduced signal above maintenance threshold. | Derived to match $C_{CNV}^{\left( Target \right)}$– see Section 4, p16 | $9.729\times{10}^{-19}\text{m}^{2}\text{day}^{-1}$ |
| $a_{2}^{(0)}$ | Starting threshold for fraction of bound VEGFR2 receptors required for maintenance of the existing choroid. | Derived by setting equal to the computationally derived fraction of bound VEGFR2 receptors in unaffected patients – see Section 4, p15. | 4.65% |
| $S_{edema}^{(ref)}$ | Scale controlling the increase in the required choroid maintenance threshold for the fraction of bound VEGFR2 receptors. | Set equal to 9 optic disc areas from clinical judgement – see Section 4, p15. | 21.6mm^2^ |
| $\theta$ | Contact angle. | Estimated from OCT images and from real patient edema width & height measurements - see Section 5, p20. | 30° |
| $S_{edema}^{\left( Target \right)}$ | Target growth rate for $S_{edema}$. | From clinical judgement based upon clinical sample of affected patients – see Section 5, p21. | 7.5 Disc areas per year |
| $\sigma_{edema}r_{edema}$ | Rate of fluid clearance per unit area of the base of the edema. | From literature – Stamer et al. (2003) | $4\mu\text{L}\text{cm}^{-2}\text{h}^{-1}$ |
| $\sigma_{C}r_{C}$ | Rate of fluid exudation per unit area of the ingressed choroid. | $\sigma_{edema}r_{edema}\times\frac{S_{edema}^{(Target)}}{C_{CNV}^{(Target)}}$  See Eq.(S.24), Section 5, p21 | $43.6\mu\text{L}\text{cm}^{-2}\text{h}^{-1}$ |

# Sensitivity of model output to variation in parameter values

In order to assess the impact of any variation in parameters we performed a sensitivity analysis – namely, we quantify the effect on a chosen end-point metric, e.g. maximum untreated edema height reached, when we make a small change in a specified parameter. As there are an infinite number of such end-point metrics, any choice is necessarily subjective. We have chosen two end-point metrics which reflect the edema growth aspects of the model, and two end-point metrics which reflect the response to treatment aspects of the model. The metrics we have chosen are, i) maximum untreated edema height, ii) untreated edema height at 1yr after onset of ischemia, iii) minimum edema height after a single standard injection at 1 month after onset of edema growth, iv) edema height two weeks after a single standard injection at 1 month after onset of edema growth. For each end-point metric and parameter we can define an elasticity, which is the instantaneous fractional change in the end-point metric per unit fractional increase in the parameter. Thus for metric $m_{i}$, and parameter $\beta_{j}$, the elasticity $\varepsilon_{ij}$ is defined as,

$$\varepsilon_{ij}= \frac{\beta_{j}}{m_{i}}\frac{\partial m_{i}}{\partial\beta_{j}} = \frac{\partial\log m_{i}}{\partial\log\beta_{j}} \text{Eq.}\left( \text{S.25} \right)$$

Consequently, an elasticity of 0.5, for example, would mean that a 1% relative increase in the parameter value will result in a 0.5% relative increase in the end-point metric value. The derivatives in Eq.(S.25) are evaluated at the baseline point, i.e. with all parameters set to their values given in Table 2. We obtain elasticity estimates by numerical differentiation – for each parameter we repeat the model simulation (including estimation of $v_{0}N_{RPE}\left( 0 \right), v_{1}N_{RPE}(0)$ and $a_{2}^{(0)} )$ using a perturbed parameter value that is 0.5% relatively higher than its baseline value given in Table 2, and again using a perturbed parameter value that is 0.5% relatively lower than its baseline value given in Table 2.

It should be noted that not all the parameters given in Table 2 are independent of each other. For example, as suggested above the VEGF production rate, $v_{0}N_{RPE}\left( 0 \right)$, required for homeostasis of the existing choriocapillaris in non-ischemic patients will be dependent upon a number of the other model parameters, e.g. the VEGF to VEGF receptor binding rate. Likewise the clearance rate $\kappa_{3}$ is dependent upon the molecular weight of the VEGF and anti-VEGF molecular species. Therefore, we have only obtained elasticity estimates for the parameters that are independent of each other. The elasticity estimates are given in Table 3. For the parameter $\phi$ the baseline value is zero. Consequently, we report instead elasticity estimates with respect to $1-\phi$.

Table 3: Elasticity estimates for all parameters.

| **Parameter** | **Definition** | **Elasticity of maximum untreated edema height** | **Elasticity of untreated edema height at 1 year** | **Elasticity of minimum edema height post injection** | **Elasticity of edema height at 2 weeks post injection** |
| --- | --- | --- | --- | --- | --- |
| $v_{I}$ | Mass of anti-VEGF agent introduced into 1^st^ compartment due to a single injection. | 0.00 | 0.00 | -$4.57\times{10}^{-2}$ | -$2.21\times{10}^{-4}$ |
| $\omega_{0}$ | Typical vitreal concentration of VEGF in unaffected patients. | -$0.26$ | -$0.29$ | -$0.94$ | -$0.62$ |
| $\omega_{1}$ | Typical vitreal concentration of VEGF in affected patients. | $0.21$ | $0.20$ | $0.74$ | $0.48$ |
| $\tau_{1}$ | Reciprocal of rate at which healthy RPE cells become ischemic and move to an elevated rate of VEGF production. | $8.14\times{10}^{-2}$ | $9.65\times{10}^{-3}$ | -$0.25$ | -$0.16$ |
| $\tau_{2}$ | Reciprocal of rate at which ischemic RPE cells cease elevated VEGF production. | $7.95\times{10}^{-2}$ | $7.16\times{10}^{-3}$ | -$0.16$ | -$0.11$ |
| $\tau_{lag}$ | Lag between elevated VEGF production and initiation of neovascularization. | -$9.17\times{10}^{-2}$ | -$9.17\times{10}^{-2}$ | $8.54\times{10}^{-2}$ | $7.16\times{10}^{-2}$ |
| $1-\phi$ | 1 - Ratio of post-ischemic RPE cell VEGF production rate to pre-ischemic RPE cell VEGF production rate. | -$5.99\times{10}^{-3}$ | -$3.66 \times{10}^{-4}$ | $7.55 \times{10}^{-3}$ | $5.02\times{10}^{-3}$ |
| $M_{\text{VEGF}}$ | Molecular weight of VEGF. | $5.81\times{10}^{-2}$ | $8.89\times{10}^{-2}$ | $0.20$ | $0.14$ |
| $M_{\text{anti-VEGF}}$ | Molecular weight of anti-VEGF. | 0.00 | 0.00 | $4.79\times{10}^{-2}$ | $2.26\times{10}^{-4}$ |
| $\kappa_{1}$ | Clearance rate of VEGF from first compartment. | -$5.04\times{10}^{-2}$ | -$5.04\times{10}^{-2}$ | -$8.40\times{10}^{-2}$ | -$7.78\times{10}^{-2}$ |
| $\kappa_{2}$ | Clearance rate of anti-VEGF from first compartment. | 0.00 | 0.00 | $0.31$ | $1.63\times{10}^{-5}$ |
| $d_{1}$ | Binding constant for binding of VEGF and anti-VEGF. | 0.00 | 0.00 | -$4.37\times{10}^{-2}$ | -$8.62\times{10}^{-4}$ |
| $d_{-1}$ | Dissociation constant for unbinding of VEGF from anti-VEGF. | 0.00 | 0.00 | $1.94\times{10}^{-2}$ | $4.97\times{10}^{-5}$ |
| $d_{2}$ | Binding constant for binding of VEGF and VEGF receptors. | -$5.26\times{10}^{-2}$ | -$8.51\times{10}^{-2}$ | -$0.23$ | -$0.16$ |
| $d_{-2}$ | Dissociation constant for unbinding of VEGF from VEGF receptors. | $5.26\times{10}^{-2}$ | $8.50\times{10}^{-2}$ | $0.24$ | $0.16$ |
| $R$ | Radius of vitreous. | -$0.12$ | -$0.18$ | -$0.18$ | -$0.25$ |
| $S_{Disc}$ | Area of optic disc. | $0.56$ | $0.58$ | $0.66$ | $0.63$ |
| $S^{(c)}$ | Surface area of interface between first and second compartments. | $5.54\times{10}^{-2}$ | $8.49\times{10}^{-2}$ | $0.18$ | $0.14$ |
| $H^{(2)}$ | Height of second compartment. | $1.56\times{10}^{-3}$ | -$1.12\times{10}^{-3}$ | -$4.44\times{10}^{-2}$ | -$2.07\times{10}^{-2}$ |
| PF | Cross-sectional packing fraction of choriocapillaris capillaries within the second compartment. | $2.07\times{10}^{-3}$ | -$7.46\times{10}^{-4}$ | -$3.37\times{10}^{-2}$ | -$2.36\times{10}^{-2}$ |
| $R_{C}$ | Typical capillary radius within the 2^nd^ compartment. | $1.33\times{10}^{-3}$ | $5.26\times{10}^{-3}$ | $3.42\times{10}^{-2}$ | $2.44\times{10}^{-2}$ |
| $\sigma_{R}$ | Density (per unit area) of VEGFR2 receptors on the surface of choroid endothelial cells. | $5.51\times{10}^{-3}$ | $3.81\times{10}^{-3}$ | -$3.32\times{10}^{-2}$ | -$2.26\times{10}^{-2}$ |
| $b_{0}$ | Proportion of capillary surface area in choriocapillaris that acts as potential source of CNV ingress into sub-retinal space. | -$3.43\times{10}^{-3}$ | -$4.55\times{10}^{-3}$ | -$5.07\times{10}^{-4}$ | -$9.31\times{10}^{-4}$ |
| $C_{CNV}^{\left( Target \right)}$ | Target CNV ingress growth rate | $3.44\times{10}^{-3}$ | $4.55\times{10}^{-3}$ | $5.27\times{10}^{-4}$ | $9.23\times{10}^{-4}$ |
| $S_{edema}^{(ref)}$ | Scale controlling the increase in the required choroid maintenance threshold for the fraction of bound VEGFR2 receptors. | $0.38$ | $0.31$ | $0.11$ | $7.47\times{10}^{-2}$ |
| $\theta$ | Contact angle. | $1.05$ | $1.05$ | $1.05$ | $1.05$ |
| $S_{edema}^{\left( Target \right)}$ | Target growth rate for $S_{edema}$. | $0.12$ | $0.19$ | $0.39$ | $0.43$ |
| $\sigma_{edema}r_{edema}$ | Rate of fluid clearance per unit area of the base of the edema. | -$3.48\times{10}^{-4}$ | -$2.10\times{10}^{-4}$ | -$1.37\times{10}^{-3}$ | -$2.36\times{10}^{-3}$ |

References:

1. Schmidt-Erfurth U, Kaiser PK, Korobelnik JF, et al. Intravitreal Aflibercept Injection for Neovascular Age-Related Macular Degeneration: Ninety-Six-Week Results of the View Studies. *Ophthalmology* 2014;121:193-201.

2. Ramrattan RS, van der Schaft TL, Mooy CM, de Bruijn WC, Mulder PG, de Jong PT. Morphometric Analysis of Bruch's Membrane, the Choriocapillaris, and the Choroid in Aging. *Invest Ophthalmol Vis Sci* 1994;35:2857-64.

3. Moshfeghi DM, Blumenkranz MS. Role of Genetic Factors and Inflammation in Age-Related Macular Degeneration. *Retina* 2007;27:269-75.

4. Quigley HA, Brown AE, Morrison JD, Drance SM. The Size and Shape of the Optic Disc in Normal Human Eyes. *Arch Ophthalmol* 1990;108:51-7.

5. Meyer CH, Holz FG. Preclinical Aspects of Anti-Vegf Agents for the Treatment of Wet Amd: Ranibizumab and Bevacizumab. *Eye* 2011;25:661-72.

6. Funk M, Kriechbaum K, Prager F, et al. Intraocular Concentrations of Growth Factors and Cytokines in Retinal Vein Occlusion and the Effect of Therapy with Bevacizumab. *Invest Ophthalmol Vis Sci* 2009;50:1025-32.

7. Noma H, Funatsu H, Yamasaki M, et al. Aqueous Humour Levels of Cytokines Are Correlated to Vitreous Levels and Severity of Macular Oedema in Branch Retinal Vein Occlusion. *Eye* 2008;22:42-8.

8. Stefanini MO, Wu FT, Mac Gabhann F, Popel AS. Increase of Plasma Vegf after Intravenous Administration of Bevacizumab Is Predicted by a Pharmacokinetic Model. *Cancer Res* 2010;70:9886-94.

9. Papadopoulos N, Martin J, Ruan Q, et al. Binding and Neutralization of Vascular Endothelial Growth Factor (Vegf) and Related Ligands by Vegf Trap, Ranibizumab and Bevacizumab. *Angiogenesis* 2012;15:171-85.

10. Yang J, Wang X, Fuh G, et al. Comparison of Binding Characteristics and in Vitro Activities of Three Inhibitors of Vascular Endothelial Growth Factor A. *Mol Pharm* 2014;11:3421-30.

11. Stefanini MO, Wu FT, Mac Gabhann F, Popel AS. A Compartment Model of Vegf Distribution in Blood, Healthy and Diseased Tissues. *BMC Syst Biol* 2008;2:77.

12. Venturoli D, Rippe B. Ficoll and Dextran Vs. Globular Proteins as Probes for Testing Glomerular Permselectivity: Effects of Molecular Size, Shape, Charge, and Deformability. *Am J Physiol Renal Physiol* 2005;288:F605-13.

13. Ferrara N, Gerber HP, LeCouter J. The Biology of Vegf and Its Receptors. *Nat Med* 2003;9:669-76.

14. Park SJ, Choi Y, Na YM, et al. Intraocular Pharmacokinetics of Intravitreal Aflibercept (Eylea) in a Rabbit Model. *Invest Ophthalmol Vis Sci* 2016;57:2612-7.

15. Stewart MW. What Are the Half-Lives of Ranibizumab and Aflibercept (Vegf Trap-Eye) in Human Eyes? Calculations with a Mathematical Model. *Eye Reports* 2011;1:12-14.

16. Del Amo EM, Urtti A. Rabbit as an Animal Model for Intravitreal Pharmacokinetics: Clinical Predictability and Quality of the Published Data. *Exp Eye Res* 2015;137:111-24.

17. Klettner A. Vegf-a and Its Inhibitors in Age-Related Macular Degeneration – Pharmacokinetic Differences and Their Retinal and Systemic Implications. *Journal of Biochemical and Pharmacological Research* 2014; 2:8-20

18. Bron AJ, Tripathi RC, Tripathi BJ. Wolff's Anatomy of the Eye and Orbit, eighth ed. London: Chapman &Hall, 1997.

19. Imoukhuede PI, Popel AS. Expression of Vegf Receptors on Endothelial Cells in Mouse Skeletal Muscle. *PLoS One* 2012;7:e44791.

20. Chen JH, Wang XC, Kan M, Sato JD. Effect of Fgf-1 and Fgf-2 on Vegf Binding to Human Umbilical Vein Endothelial Cells. *Cell Biol Int* 2001;25:257-60.

21. Florey HW. The Endothelial Cell. *Br Med J* 1966;2:487-90.

22. Garipcan B, Maenz S, Pham T, et al. Image Analysis of Endothelial Microstructure and Endothelial Cell Dimensions of Human Arteries – a Preliminary Study. *Advanced Engineering Materials* 2011;13: B54–B57.

23. Milo R, Jorgensen P, Weber G, Springer M. Bionumbers—the Database of Key Numbers in Molecular and Cell Biology. *Nucleic Acids Res* 2010;38:D750-753.

24. Staurenghi G, Orzalesi N, La Capria A, Aschero M. Laser Treatment of Feeder Vessels in Subfoveal Choroidal Neovascular Membranes: A Revisitation Using Dynamic Indocyanine Green Angiography. *Ophthalmology* 1998;105:2297-305.

25. Olsson AK, Dimberg A, Kreuger J, Claesson-Welsh L. Vegf Receptor Signalling - in Control of Vascular Function. *Nat Rev Mol Cell Biol* 2006;7:359-71.

26. Klettner A, Recber M, Roider J. Comparison of the Efficacy of Aflibercept, Ranibizumab, and Bevacizumab in an Rpe/Choroid Organ Culture. *Graefes Arch Clin Exp Ophthalmol* 2014;252:1593-8.

27. Saint-Geniez M, Kurihara T, Sekiyama E, Maldonado AE, D'Amore PA. An Essential Role for Rpe-Derived Soluble Vegf in the Maintenance of the Choriocapillaris. *Proc Natl Acad Sci USA* 2009;106:18751-6.

28. Della NG, Campochiaro PA, Zack DJ. Localization of Timp-3 Mrna Expression to the Retinal Pigment Epithelium. *Invest Ophthalmol Vis Sci* 1996;37:1921-4.

29. Strunnikova NV, Barb J, Sergeev YV, et al. Loss-of-Function Mutations in Rab Escort Protein 1 (Rep-1) Affect Intracellular Transport in Fibroblasts and Monocytes of Choroideremia Patients. *PLoS One* 2009;4:e8402.

30. Qi JH, Ebrahem Q, Moore N, et al. A Novel Function for Tissue Inhibitor of Metalloproteinases-3 (Timp3): Inhibition of Angiogenesis by Blockage of Vegf Binding to Vegf Receptor-2. *Nat Med* 2003;9:407-15.

31. Zhang SX, Wang JJ, Gao G, Parke K, Ma JX. Pigment Epithelium-Derived Factor Downregulates Vascular Endothelial Growth Factor (Vegf) Expression and Inhibits Vegf-Vegf Receptor 2 Binding in Diabetic Retinopathy. *J Mol Endocrinol* 2006;37:1-12.

32. Martin S, Favot L, Matz R, Lugnier C, Andriantsitohaina R. Delphinidin Inhibits Endothelial Cell Proliferation and Cell Cycle Progression through a Transient Activation of Erk-1/-2. *Biochem Pharmacol* 2003;65:669-75.

33. Ohno-Matsui K, Yoshida T. Myopic Choroidal Neovascularization: Natural Course and Treatment. *Curr Opin Ophthalmol* 2004;15:197-202.

34. Vander JF, Morgan CM, Schatz H. Growth Rate of Subretinal Neovascularization in Age-Related Macular Degeneration. *Ophthalmology* 1989;96:1422-6; discussion 1426-9.

35. Stamer WD, Bok D, Hu J, Jaffe GJ, McKay BS. Aquaporin-1 Channels in Human Retinal Pigment Epithelium: Role in Transepithelial Water Movement. *Invest Ophthalmol Vis Sci* 2003;44:2803-8.

36. RCoreTeam. R: A Language and Environment for Statistical Computing. Vienna, Austria: R Foundation for Statistical Computing, 2015.

37. Soetaert K, Petzoldt T, Setzer R. Solving Differential Equations in R: Package Desolve. *Journal of Statistical Software* 2010;33:1 -25.
